# Electrochemical maps and movies of the hydrogen evolution reaction on natural crystals of molybdenite (MoS_2_): basal *vs.* edge plane activity†
†Electronic supplementary information (ESI) available: Movies S1 to S4: spatially resolved LSV-SECCM movies obtained from the electrocatalytic HER on the surface of bulk MoS_2_. Fig. S1 to S14: XRD, XPS, Raman, SEM and OM characterization of MoS_2_; SEM images of the nanopipets; WCA measurements; LSVs and Tafel plots obtained from the HER on MoS_2_. See DOI: 10.1039/c7sc02545a



**DOI:** 10.1039/c7sc02545a

**Published:** 2017-07-26

**Authors:** Cameron L. Bentley, Minkyung Kang, Faduma M. Maddar, Fengwang Li, Marc Walker, Jie Zhang, Patrick R. Unwin

**Affiliations:** a Department of Chemistry , University of Warwick , Coventry CV4 7AL , UK . Email: C.Bentley.1@warwick.ac.uk ; Email: P.R.Unwin@warwick.ac.uk; b School of Chemistry , Australian Research Council Centre of Excellence for Electromaterials Science , Monash University , Clayton , Vic 3800 , Australia; c Department of Physics , University of Warwick , Coventry CV4 7AL , UK

## Abstract

Two dimensional (2D) semiconductor materials, such as molybdenum disulfide (MoS_2_) have attracted considerable interest in a range of chemical and electrochemical applications, for example, as an abundant and low-cost alternative electrocatalyst to platinum for the hydrogen evolution reaction (HER). While it has been proposed that the edge plane of MoS_2_ possesses high catalytic activity for the HER relative to the “catalytically inert” basal plane, this conclusion has been drawn mainly from macroscale electrochemical (voltammetric) measurements, which reflect the “average” electrocatalytic behavior of complex electrode ensembles. In this work, we report the first spatially-resolved measurements of HER activity on natural crystals of molybdenite, achieved using voltammetric scanning electrochemical cell microscopy (SECCM), whereby pixel-resolved linear-sweep voltammogram (LSV) measurements have allowed the HER to be visualized at multiple different potentials to construct electrochemical flux movies with nanoscale resolution. Key features of the SECCM technique are that characteristic surface sites can be targeted and analyzed in detail and, further, that the electrocatalyst area is known with good precision (in contrast to many macroscale measurements on supported catalysts). Through correlation of the local voltammetric response with information from scanning electron microscopy (SEM) and atomic force microscopy (AFM) in a *multi-microscopy approach*, it is demonstrated unequivocally that while the basal plane of bulk MoS_2_ (2H crystal phase) possesses significant activity, the HER is greatly facilitated at the edge plane (*e.g.*, surface defects such as steps, edges or crevices). Semi-quantitative treatment of the voltammetric data reveals that the HER at the basal plane of MoS_2_ has a Tafel slope and exchange current density (*J*_0_) of ∼120 mV per decade and 2.5 × 10^–6^ A cm^–2^ (comparable to polycrystalline Co, Ni, Cu and Au), respectively, while the edge plane has a comparable Tafel slope and a *J*_0_ that is estimated to be more than an order-of-magnitude larger (∼1 × 10^–4^ A cm^–2^). Finally, by tracking the temporal evolution of water contact angle (WCA) after cleavage, it is shown that cathodic polarization has a ‘self-cleaning’ effect on the surface of MoS_2_, consistent with the time-independent (*i.e.*, time after cleavage) HER voltammetric response.

## Abstract 2

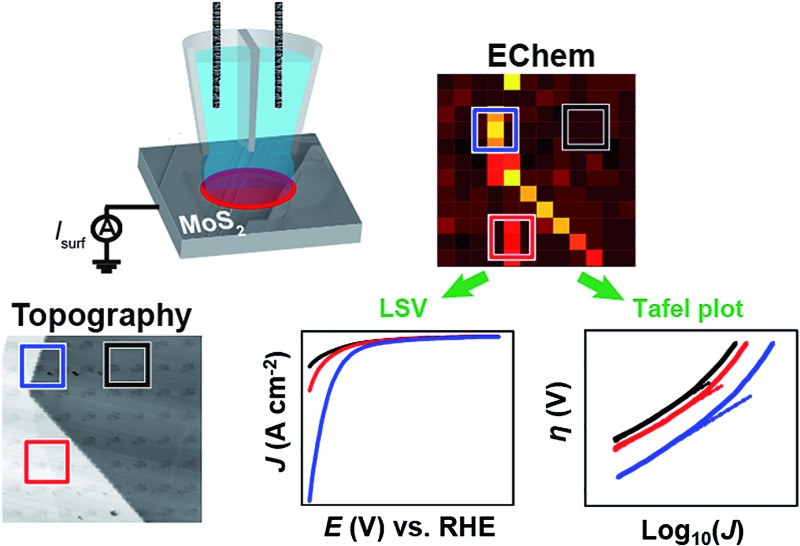
In this work, we report the first spatially-resolved voltammetric measurements of the hydrogen evolution reaction on natural crystals of molybdenite, unequivocally demonstrating enhanced catalytic activity on the edge plane relative to the basal plane.

## 1. Introduction

There is presently considerable interest in two-dimensional (2D) materials such as molybdenum disulfide (MoS_2_) for a variety of chemical and electrochemical applications.^1–3^ MoS_2_ and several of the related transition metal dichalcogenides (TMDCs) are particularly interesting because they are semiconducting, with sizable bandgaps (1–2 eV), allowing them to be utilized in a range of electronic, optoelectronic and photovoltaic devices.^4–7^ Bulk MoS_2_ possesses a layered structure in the form S–Mo–S (*i.e.*, close packed S-planes sandwiching a Mo-layer) and adjacent layers are weakly interacting (van der Waals gap = 6.15 Å), resulting in a bulk crystal that can be readily exfoliated to form atomically thin layers (*i.e.*, single unit cell thickness).^1–3,8^ The main focus herein is the application of MoS_2_ as an abundant and low-cost alternative electrocatalyst to platinum^9,10^ for the hydrogen evolution reaction (HER), a process of growing importance as the world strives towards the implementation of clean, sustainable and affordable energy technologies.^1,9,11^

Early studies by Tributsch and Bennett^12^ on bulk crystals of MoS_2_ suggested that it was not an efficient HER catalyst, but in 2005, Hinnemann and co-workers^13^ published a groundbreaking study suggesting that nanostructuring MoS_2_ can significantly improve the HER catalytic activity, based on the nearly thermoneutral hydrogen adsorption free energy (Δ*G*_H_) calculated for the Mo-edge. This was confirmed experimentally in 2007, when Jaramillo and co-workers^14^ found that the catalytic activity of MoS_2_ nanoparticles [supported on an Au(111) surface] scaled linearly with nanoparticle perimeter (edge) length, rather than surface area. Since then, the general consensus has been that the edges of MoS_2_ (2H phase) are catalytically active, whereas the basal plane is “catalytically inert” for the HER, with experimental^14–18^ and theoretical^13,19,20^ studies published by a number of research groups in support of this. For this reason, a number of studies have focused on engineering the surface structure of MoS_2_ to preferentially expose the active edge sites, for example vertically aligned layers (*i.e.*, basal plane perpendicular to the support)^21^ or double gyroid mesoporous nanostructures.^15^ In addition to increasing the density of active edge sites, recent works have shown that the basal plane of 2H MoS_2_ can be “activated” by introducing^22,23^ and further straining^18,24^ sulfur vacancies. Furthermore, crystallite quality^22^ and crystal phase^23,25,26^ has also been shown to play a crucial role, with the 1T (metallic) phase of MoS_2_ displaying superior catalytic activity to that of the corresponding 2H (semiconducting) phase.

HER catalysis studies are generally carried out with nanostructured mono/few layer MoS_2_ synthesized by various methods^2^ (*e.g.*, chemical vapour deposition,^18^ solvothermal growth,^16,27^ electrodeposition^28^ or wet chemical synthesis^29^), or produced from the bulk material by liquid-phase exfoliation.^25^ Given the reportedly poor catalytic activity of the bulk material,^9,12,19^ it is unsurprising that studies on the (electro)catalytic performance of natural crystals of MoS_2_ are scarce.^12,30–32^ Tan and co-workers^30^ recently reported that while the edge plane of bulk MoS_2_ catalyzed the HER, with activity dependent upon the surface state (*i.e.*, oxidative/reductive pre-treatment) of the crystal, the basal plane was essentially inert, showing activity that was even lower than that of glassy carbon (GC).

The catalytic performance of nanomaterials, for example MoS_2_, is usually evaluated from the “total electrode activity” measured with macroscopic (“bulk”) electrochemical techniques. In this approach, the (nanostructured) catalyst is immobilized on a supposedly inert, conducting substrate and the catalytic current is measured as a function of potential using voltammetry. Taking this approach, the intrinsic activity of each catalytic site (*i.e.*, the turnover frequency) can only be inferred from the total electrode activity if the number of active sites is known, which is challenging to measure for functional catalysts.^1,33^ In contrast, high resolution electrochemical imaging techniques are able to investigate and map the electrochemical activity of complex materials at the nanoscale, and target particular characteristic features on a surface.^34,35^ In particular, by combining high resolution electrochemical imaging data with information from other imaging and spectroscopic techniques applied to the same area in a *correlative multi-microscopy approach*, micro/nanoscopic structure (*i.e.*, basal *vs.* edge plane) can be unequivocally related to function (*i.e.*, catalytic activity), which is the ultimate goal in materials science.^33,36^ Thus, using high resolution electrochemical imaging techniques such as scanning electrochemical cell microscopy (SECCM), our group has conclusively shown that the basal plane of highly-oriented pyrolytic graphite (HOPG) is highly electrochemically active for several classes of redox reactions, despite the long held belief that it possessed low or no activity.^36–38^

In this work, the electrocatalytic activity of natural crystals of MoS_2_ towards the HER is mapped for the first time on the nanoscale using voltammetric SECCM.^37,39,40^ Bulk MoS_2_ was investigated because it possesses a well-defined surface that can be easily renewed by mechanical exfoliation. Pixel-resolved linear-sweep voltammogram (LSV) measurements allowed the HER to be visualized across the MoS_2_ surface at multiple different potentials that could be played back as electrochemical flux movies. The local voltammetric response was correlated to micro/nanoscopic structure (*i.e.*, basal *vs.* edge plane) by combining the electrochemical images with information from scanning electron microscopy (SEM) and atomic force microscopy (AFM). The basal plane of bulk MoS_2_ was found to support the HER in acidic media (*i.e.*, it is not “catalytically inert”, as previously reported^30^) and the presence of surface disorder (ranging from single to multiple edge plane steps, defects or crevices) enhances the kinetics of this inner-sphere (electrocatalytic) process.

## 2. Experimental

### 2.1 Chemical reagents and electrode materials

Perchloric acid (HClO_4_, Sigma-Aldrich, 70%), potassium chloride (KCl, Sigma-Aldrich) and dichlorodimethylsilane [Si(CH_3_)_2_Cl_2_, Acros Organics, ≥99%] were used as supplied by the manufacturer. All solutions were prepared with deionized water (Integra HP, Purite, U.K.), which had a resistivity of 18.2 MΩ cm (25 °C).

The naturally-occurring molybdenite (MoS_2_) crystal [semiconducting 2H phase, as characterized by Raman spectroscopy, X-ray diffraction (XRD) and X-ray photoelectron spectroscopy (XPS),^41^ see ESI, Fig. S1†] was purchased from Manchester Nanomaterials Ltd (U.K.). Prior to use as an electrode material, flakes of MoS_2_ were fixed in place using carbon SEM tape on a glass microscope slide and mechanically cleaved using the “scotch-tape method”.^42^ In order to avoid possible issues arising from ohmic resistance (through bottom-contact), the freshly-cleaved MoS_2_ flakes were electrically connected through top-contact with a steel pin. The platinum substrate was prepared by sputtering onto a glass microscope slide. The glassy carbon substrate was purchased from HTW-Germany, and polished with an aqueous slurry of 0.05 μm Al_2_O_3_ (Buehler, U.S.A.) prior to use. Silver–silver chloride (Ag/AgCl) quasi-reference counter electrodes (QRCEs) were prepared by anodizing 0.125 mm diameter annealed silver wire (Goodfellow, U.K., 99.99%) in a saturated solution of KCl. The QRCEs were calibrated potentiometrically in the solution of interest (*i.e.*, 5 or 100 mM HClO_4_) against a commercial saturated calomel electrode (SCE), which has a potential of +0.241 V *vs.* the standard hydrogen electrode (SHE).^43^

### 2.2 Material characterization

Optical microscopy was carried out with a BH-2 optical microscope (Olympus, Japan). Optical micrographs were captured using a SPOT Idea 5Mp camera (Diagnostic Instruments Inc., U.S.A.) using the SPOT Imaging Software (v. 4.7, Diagnostic Instruments Inc.). Field emission SEM images of the nanopipet tips and MoS_2_ substrates were obtained on a SUPRA 55-VP and GeminiSEM 500 scanning electron microscope (Zeiss, Germany), respectively, at acceleration voltages in the range 0.5 to 3 kV, with an InLens detector. AFM was carried out in tapping mode using silicon probes with a spring constant of 3 N m^–1^ according to manufacturer (RFESP, Bruker, Germany), on an Innova atomic force microscope (Bruker). AFM image processing was carried out with the scanning probe image processing software package (SPIP v. 6.0.14, Image Metrology, Denmark).

Contact angle measurements were carried out as previously reported.^44^ In brief, a 10 μL droplet of water was gently placed atop a bulk MoS_2_ surface, and optical images of the droplet (side view) were recorded with a digital camera (PixeLINK PL-B782U, equipped with a 2× magnification lens, 1920 × 1080 pixels). The resulting images were analyzed (6 to 10 measurements were made under each experimental condition) using the ImageJ software package (1.49v, National Institutes of Health, U.S.A.) with the contact angle for the droplet being measured at the contact line.

XRD was performed on a D2 PHASER powder diffractometer (Bruker, Germany) with a Cu Kα radiation source (*λ* = 0.1541 nm). Raman spectroscopy was carried out on an inVia Microscope (Renishaw, U.K.) with a 514 nm laser source. XPS was carried out on an Axis Ultra DLD Spectrometer (Kratos Analytical, Japan) with an Al Kα radiation source (*λ* = 0.8340 nm).

### 2.3 Electrochemical experiments

All experiments were carried out at room temperature (22 ± 2 °C). Conventional electrochemical experiments were performed in a three electrode format with an Ag/AgCl QRCE (preparation described above) and platinum wire (Goodfellow, U.K.) auxiliary electrode on a CHI-730A potentiostat (CH instruments, U.S.A.). All other electrochemical experiments were carried out in the SECCM format on a home-built electrochemical workstation.^34,45^ In this setup, a dual-barreled nanopipet probe was filled with electrolyte solution (5 or 100 mM HClO_4_) and mounted on a *z*-piezoelectric positioner (P-753.3CD, PhysikInstrumente). The tip of the nanopipet probes were elliptical in shape, with major (*r*_a_) and minor (*r*_b_) radii of approximately 250 nm and 130 nm, respectively, as shown in Fig. S2a.† Ag/AgCl wire placed in each barrel served as QRCEs (detailed above). A bias potential (*E*_b_) of either +0.05 V (100 mM HClO_4_) or +0.2 V (5 mM HClO_4_) was applied between the QRCEs in order to generate an ion conductance current, which was used as a feedback signal during positioning of the nanopipet probe (see below). The nanopipet was positioned above the surface of interest using micropositioners for coarse movement and an *xy*-piezoelectric positioner (P-622.2CD, PhysikInstrumente) for fine movement. The nanopipet was oscillated normal to the surface of interest (*f* ≈ 280 Hz, Δ*z* ≈ 30 nm peak-to-peak) by an ac signal generated by a lock-in amplifier (SR830, Stanford Research Systems, U.S.A.) applied to the *z*-piezoelectric positioner. During approach, the magnitude of the ac ion conductance current generated by distance modulation (measured using the same lock-in amplifier) was used as feedback to detect when the meniscus at the end of the nanopipet had made contact with the working electrode surface.^34,45^ The nanopipet itself did not contact the substrate. Electrochemical (voltammetric) measurements were performed in the confined area defined by the meniscus (droplet cell) created between the tip and substrate. The size of the confined area (*i.e.*, working electrode area) was determined by (SEM) imaging the droplet “footprint” left after electrochemical measurements, as demonstrated in Fig. S2b.†

**Fig. 1:**
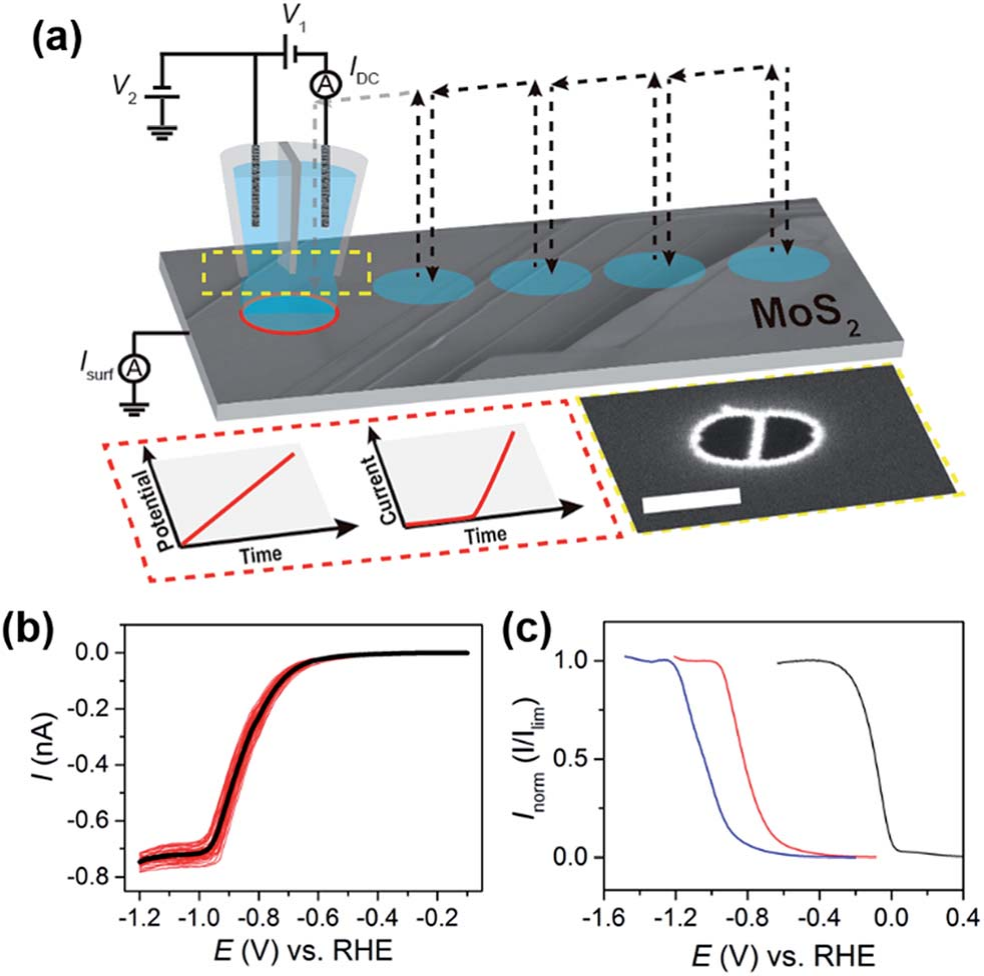
(a) Schematic showing the nanoscopic electrocatalytic measurements made using voltammetric hopping mode SECCM. For SECCM, a bias voltage of *V*_1_ was applied between the two Ag/AgCl wire QRCEs and the resulting ion conductance current (*I*_dc_) was measured and employed for probe positioning. A substrate voltage of *V*_2_ was applied to one of the QRCEs to control the working electrode (*e.g.*, MoS_2_) potential (*E*_s_), where *E*_s_ = –(*V*_1_/2 + *V*_2_), and the working electrode current (*I*_surf_) was measured (*E*–*t* and *I*–*t* wave-forms shown inset). The arrows show the movement of the nanopipet probe along the surface. Also shown in the inset is an SEM image of the end of a representative nanopipet probe (the scale bar indicates 400 nm). (b) Individual (red) and averaged (black) LSVs obtained from 38 different points on the basal plane of bulk MoS_2_ using the experimental setup outlined in (a). (c) Normalized LSVs obtained on (from left to right) bulk GC (blue trace), MoS_2_ basal plane (red trace), and Pt (black trace). All LSVs were obtained using the following experimental parameters: [HClO_4_] = 5 mM, *ν* = 0.5 V s^–1^, *E*_b_ = +0.2 V, *r*_a_ = 275 nm and *r*_b_ = 125 nm.

Electrochemical measurements at the substrate (working electrode) were made using a linear-sweep voltammetric “hopping” regime, as described previously.^37,39,40^ In brief, as shown schematically in Fig. 1a, the nanopipet was approached to the surface of interest at a series of predefined locations in a grid and, upon each landing, a linear sweep voltammetric experiment was carried out, building up an voltammetric ‘map’ of the substrate. In other words, in the resulting “electrochemical map” (equipotential image), each pixel corresponds to an individual LSV. The hopping distance between each pixel was 1 μm to avoid overlap of the probed areas. Note that in the images and movies presented, there is no interpolation of the data.

The SECCM cell and all piezoelectric positioners were placed in an aluminum Faraday cage equipped with heat sinks and vacuum panels to block out all light (important in the study of semiconducting materials) and minimize noise and thermal drift. The QRCE potentials were controlled (with respect to ground) with a home-built bipotentiostat and the substrate (working electrode, common ground) current was measured using a home-built electrometer with variable data acquisition times. A home-built 16th order (low-pass) brick-wall filter unit (time constant = 2 ms) was utilized during data (current) collection. Data acquisition and fine control of all the instruments was achieved using an FPGA card (PCIe-7852R) controlled by a LabVIEW 2016 (National Instruments, U.S.A.) interface. Data treatment and analysis was carried out using the Matlab R2015b (8.6.0.267246, Mathworks, U.S.A.) and OriginPro 2016 64bit (b9.3.226, OriginLab, U.S.A.) software packages.

The dual-barrelled nanopipets were pulled from quartz filamented theta-capillaries (QTF120-90-100, Friedrich & Dimmock Inc., U.S.A.) using a CO_2_-laser puller (P-2000, Sutter Instruments, U.S.A.). Following pulling, the outer walls of nanopipet tips were silanized with dichlorodimethylsilane to aid meniscus confinement (and stability) when coming into contact with the substrate of interest. After the nanopipet tips were filled with the solution of interest using a MicroFil syringe (World Precision Instruments Inc., U.S.A.), a layer of silicone oil (DC 200, Sigma-Aldrich) was added on top in order to minimize evaporation (exacerbated by the filament, shown schematically in Fig. S3†). The QRCEs were then inserted through the oil layer, into the solution of interest, and mounted on the *z*-piezoelectric positioner, as described above.

## 3. Results and discussion

### 3.1 Voltammetric characteristics of the HER on MoS_2_ at low [H^+^]

The HER is postulated to proceed *via* the Volmer reaction (eqn (1)), followed by either the Tafel (eqn (2)) or Heyrovsky (eqn (3)) reaction in acidic aqueous media:^10,46^

1 H^+^ + e^–^ → H_ads_

2 2H_ads_ → H_2_

3 H^+^ + H_ads_ + e^–^ → H_2_

where H_ads_ is an adsorbed hydrogen atom. Thus, following the formation of H_ads_ on the electrode surface (eqn (1)), H_2_ is formed either through dimerization of H_ads_ (eqn (2)) or directly from H_ads_ upon electron transfer to H^+^ in solution (eqn (3)). The preferred reaction pathway depends on the nature of the electrode material,^10^ and is not yet fully understood on MoS_2_ (reported Tafel slopes vary depending on synthesis conditions and support^1^), although the mechanism has been the subject of a number of recent theoretical studies.^19,20^

The steady-state mass-transport limited current, *I*_lim_, depends on the geometry of the nanopipet and active electrode area (*i.e.*, the meniscus cell), and therefore acts as a reliable indicator of the wetting of the droplet on the MoS_2_ surface. For this reason, the concentration of acid was initially kept low (5 mM or pH 2.3), so that the entire HER reduction wave could be fully investigated. The voltammetric characteristics of the HER were elucidated on a natural crystal of MoS_2_ using the SECCM format,^34,45^ as shown schematically in Fig. 1a. Initially, sub-millimeter pieces of the exfoliated crystal were physisorbed onto a GC substrate, as demonstrated in the optical micrograph shown in Fig. S4a.† Due to the semiconducting nature of MoS_2_, it was found that the crystals prepared in this manner either gave rise to no electrochemical signal (*i.e.*, there was no electrical connection) or LSVs displaying severe ohmic distortion, as shown in Fig. S4b.† In order to overcome this limitation, for the SECCM studies herein, the electrical connection was made through top contact with a steel pin, taking advantage of the fact that in-plane electron mobility (*i.e.*, parallel to the basal plane) of bulk MoS_2_ is more than three orders of magnitude higher than out-of-plane (*i.e.*, perpendicular to the basal plane).^1,47^

Representative LSVs showing the HER, recorded from 5 mM HClO_4_ with a scan rate of 0.5 V s^–1^ at 38 different points on the MoS_2_ crystal (see Fig. S5†) are shown in Fig. 1b. The proton reduction wave is sigmoidal in shape, indicating near steady-state conditions, with a half-wave potential (*E*_1/2_) of approximately –0.85 V *vs.* RHE. Overall the proton reduction waves are relatively homogeneous, with *E*_1/2_ values varying by less than 50 mV from point-to-point (the relationship between *E*_1/2_ and surface structure is discussed in detail below). Clearly, *I*_lim_ is well-defined, but varies slightly from point-to-point, ranging from –0.70 to –0.78 nA in Fig. 1b. Taking into account that the diffusional flux in SECCM is approximately 10% of that for the same sized disk electrode,^45^ and the diffusion coefficient of the hydronium ion (H_3_O^+^) is 9.3 × 10^–5^ cm^2^ s^–1^ in aqueous media,^48^ a steady-state limiting current of *ca.* –0.6 nA is expected from a droplet of the dimensions shown in Fig. S2b,† assuming that mass-transport occurs solely by diffusion. This is slightly lower than what is observed experimentally (≈–0.74 nA, see Fig. 1b), attributable to effects of migration, which is expected to make a non-negligible contribution to the flux of H^+^ (or H_3_O^+^) under the conditions outlined in Fig. 1 (*i.e.*, *E*_b_ = +0.2 V and no supporting electrolyte).^45^

The kinetics of the HER can be readily evaluated (qualitatively) from near steady-state LSV data (*e.g.*, see Fig. 1b), based on the position of the wave on the potential-axis (*i.e.*, *E*_1/2_).^43^ This is demonstrated in Fig. 1c, which compares the HER voltammetric response (in the SECCM format, see Fig. 1a) obtained from Pt, GC and bulk MoS_2_. It should be noted that no special precautions were taken to clean the sputtered (nanocrystalline) Pt surface prior to use (*e.g.*, by flame annealing), and has simply been included as a qualitative comparison. The data shown in Fig. 1c are normalized with respect to *I*_lim_ in order to account for slight differences in the meniscus cell morphology or “active electrode area” between surfaces. This rules out a quantitative analysis of these data, additionally because the electrochemical process depletes the concentration of protons significantly, so that there is some ohmic contribution to the voltammetric response. Nonetheless, the catalytic activity of the bulk materials can be ranked and increases in the order GC < MoS_2_ ≪ Pt. Clearly, while the basal plane of bulk MoS_2_ is not comparable to Pt as an electrocatalyst for the HER (explored below), the activity is much higher than previously assumed. For example, the response on the MoS_2_ basal surface is significantly better than GC, which is at odds with an earlier report by Tan and co-workers.^30^ The extremely poor electrocatalytic response observed in that study is likely attributable to high ohmic resistance as a result of the resistive nature of bulk MoS_2_ (for example, see Fig. S4†) which makes macroscale measurements extremely problematic.

### 3.2 Electrochemical (voltammetric) mapping of MoS_2_ at low [H^+^]

**Fig. 2:**
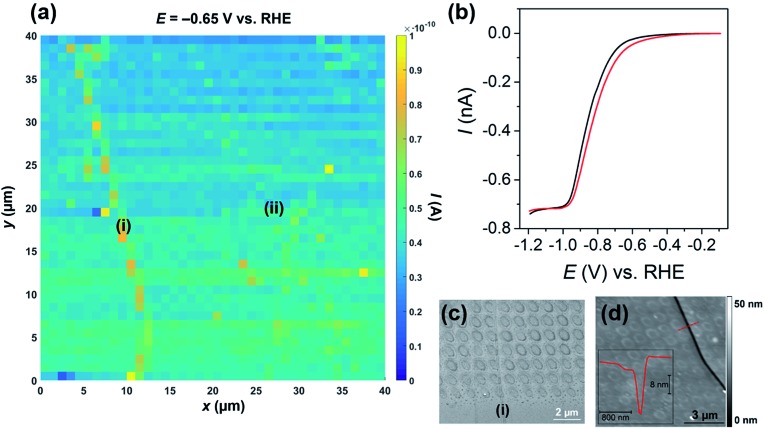
(a) 40 × 40 μm spatially resolved current map (equipotential image) obtained at –0.65 V *vs.* RHE (see Movie S1† for full potential range). Major and minor surface defects are labelled as (i) and (ii), respectively. (b) Representative LSVs obtained from areas containing only basal plane (black trace, average of 1000 measurements) and basal plus edge plane [red trace, defect (i), average of 30 measurements] of bulk MoS_2_. The following parameters were used in (a) and (b): [HClO_4_] = 5 mM, *ν* = 0.5 V s^–1^, *E*_b_ = +0.2 V, *r*_a_ = 275 nm and *r*_b_ = 125 nm. (c) SEM and (d) AFM topographical images of the scan area. Inset in (d) is an AFM line profile of the area indicated by the dashed red line.

The catalytic activity of MoS_2_ towards the HER was mapped using voltammetric SECCM, whereby the nanopipet probe was approached to the surface at a series of predefined locations (1 μm spacing) and upon each meniscus landing, an LSV was recorded. A spatially resolved LSV-SECCM movie, collected at a pH of 2.3 (*i.e.*, [H^+^] = 5 mM), is shown in the ESI, Movie S1.† The movie consists of 111 images (*i.e.*, 1 image every 10 mV), and each pixel (1600 in total) represents an individual LSV in the 40 × 40 μm scan area. A spatially resolved equipotential image (*i.e.*, current “map”), taken at –0.65 V *vs.* RHE is shown in Fig. 2a. Evidently, the activity of the basal plane surface is relatively uniform, with one obvious step “feature” or “defect” [labelled as (i) in Fig. 2a and evident from optical microscopy, as shown in Fig. S6†] giving rise to enhanced current at this potential, indicating more facile HER kinetics (explored below).

The LSVs extracted from the “defect” pixels are shifted positively with respect to the ‘average’ basal plane LSV, as demonstrated in Fig. 2b. It needs to be emphasized that the red current–potential (*I*–*E*) trace in Fig. 2b arises from predominantly the basal plane plus a small area (estimated to be <10% of the total meniscus cell area, see below) of edge plane, while the black *I*–*E* trace arises solely from the basal plane (*i.e.*, the green/blue areas in Fig. 2a). Clearly, *I*_lim_ does not vary between the two curves shown in Fig. 2b, indicating that the enhancement in current seen in Fig. 2a arises from a legitimate increase in HER kinetics due to the presence of edge plane in the probed meniscus cell area, rather than distortion of the droplet (meniscus) cell when landing on the defect. To further confirm this, the dc ion conductance current (*I*_dc_), which is an indicator of the droplet size/stability,^34,45^ was plotted for a number of lines in the scan (*y* = 10 to 15 μm in Fig. 2a), as shown in Fig. S7.† Evidently, there is little noticeable change in *I*_dc_ in the vicinity of the defect (*i.e.*, *x* = 9 to 12 μm), again indicating that the presence of the surface feature does not distort the morphology of the SECCM meniscus cell.

The nature of the surface defect described above was investigated with a combination of SEM (see Fig. 2c) and AFM (see Fig. 2d). Evidently, the majority of the LSVs were measured on the basal surface of MoS_2_, however, as shown in Fig. 2c and S8,† a small number of the droplet footprints (each footprint corresponds to an individual LSV, as discussed above) also intersect the major defect spanning the left side of the scan area [labelled (i) in Fig. 2a]. The defect is also very obvious in the AFM topographical image shown in Fig. 2d, and a line scan profile revealed that it is a ∼20 nm deep “crevice”, as shown in the inset. Interestingly, the individual droplet footprints are evident in the AFM topographical image, implying that the voltammetric protocol employed during the electrochemical scanning (*i.e.*, cathodic polarization) causes a physical (structural) change to the MoS_2_ surface. Finally, it should be noted that there are other “defects” present in the scan area, which also gave rise to slightly enhanced currents [labelled as (ii) in Fig. 2a and S9†], relative to the basal plane. These minor defects were confirmed to be single to few-layer step edges by AFM line profile scanning (data not shown). The relationship between defect morphology (*i.e.*, size or area) and the observed enhancement in HER kinetics is explored in much greater detail below.

The potential (*i.e.*, *E*_1/2_) shift observed in Fig. 2b arises from an enhancement in HER kinetics caused by the presence of surface disorder (*i.e.*, edge plane), and, while the shift appears to be small (∼35 mV), as noted above, the surface defect [*e.g.*, crevice (i) in Fig. 2a] only comprises a small portion of the “active electrode area” (*i.e.*, the area probed by the meniscus cell), evident in Fig. 2c and S8.† In other words, even when landing on a defect site, with a probe of these dimensions, the majority of the response still (ultimately) originates from the basal plane. With this in mind, it is clear that the kinetics of the HER must be significantly more facile on the edge plane (defects) relative to the basal plane.^13–20^ A similar phenomenon (*i.e.*, enhanced electrochemical activity at surface defects) was recently modeled by Güell and co-workers,^37^ who used a finite element method to quantify the enhancement in *k* ^0^ for the [Ru(NH_3_)]^2+/3+^ process on the step edges of mono/few-layer graphene. The HER is significantly more complicated than the [Ru(NH_3_)]^2+/3+^ process, as it proceeds through the formation of an adsorbed intermediate species (H_ads_, see eqn (1) to (3)) *via* an unestablished mechanism on MoS_2_ (ref. 19 and 20), making quantitative treatment of the data in this fashion difficult. Nonetheless, a semi-quantitative analysis (explored below) is possible, with the advantage of knowing the surface character through the use of SECCM.

Although the electrochemical map was obtained on freshly exfoliated MoS_2_, the possible influence of “surface aging” on the observed voltammetry needs to be discussed, as each SECCM scan took upwards of 4 hours to complete. It has been widely reported that 2D materials such as MoS_2_ and graphene are susceptible to contamination by adventitious adsorbates such as airborne hydrocarbons, which can be monitored through the temporal evolution of water wettability, after exfoliation.^49^ In addition, MoS_2_ is reportedly susceptible to slow oxidation (particularly at the edge plane) under ambient (atmospheric) conditions.^17,31,50,51^ In any case, Velicky and co-workers^31^ showed in a recent study that atmospheric aging of MoS_2_ apparently decreased the electron-transfer kinetics associated with the [Fe(CN)_6_]^3–/4–^, [Ru(NH_3_)_6_]^3+/2+^ and [IrCl_6_]^2–/3–^ redox processes.

In the present study, no significant decrease in the electrochemical activity (*i.e.*, <15 mV shift) of the basal plane was observed with time (*e.g.*, see Fig. 2a), evidenced by comparing LSVs obtained at the start (*i.e.*, <1 hour after exfoliation), middle and end (*i.e.*, aged surface, after 4 hours) of the scan, as shown in Fig. S10.† In addition, LSVs were measured on MoS_2_ surfaces at times ranging from ∼30 minutes to several days after exfoliation, and no significant correlation between apparent HER kinetics and “surface age” was observed. This relatively long timescale is similar to that over which changes in contact angle are observed^49^ (see below) and thus the electrochemical data suggest that surface aging has a minimal influence on the catalytic activity of the basal plane over this timescale. The edge plane of MoS_2_ may be more susceptible to oxidation/contamination, and consequently the influence of surface aging on the observed response cannot be ruled out.

Nevertheless, assuming water wettability is an indicator of surface cleanliness, as has been proposed,^49^ the influence of surface aging was investigated by making water contact angle (WCA) measurements. Freshly cleaved MoS_2_ was found to be mildly hydrophilic, with a WCA of 72 ± 5°, and became hydrophobic after prolonged surface aging, with a measured WCA of 98 ± 3° several weeks after cleavage, in good agreement with a previous study.^49^ However, the WCA was restored to a value of 78 ± 5° after a single voltammetric cycle in 5 mM HClO_4_ (carried out in the three electrode format, with a scan rate 0.05 V s^–1^) in the potential range +0.6 to –1.9 V *vs.* RHE, as shown in Fig. S11.† The potential drop at the (semiconductor) electrode interface is not known and will be greatly reduced, from that applied, in these measurements due to significant ohmic (*IR*) drop in the material, yet it is clear that cathodic polarization in acidic media has a “cleaning” effect on the MoS_2_ surface, which likely explains why surface aging has no apparent influence on electrocatalytic performance measured in our experiments. It should be noted that a similar *in situ* cleaning effect was employed and observed when investigating the oxygen reduction reaction (ORR) on polycrystalline platinum by SECCM.^52^

### 3.3 Electrochemical (voltammetric) mapping of MoS_2_ at high [H^+^]

The performance of HER catalyst materials (*e.g.*, MoS_2_) is usually evaluated by supporting them on an electrochemically inert, conducting substrate (*e.g.*, carbon support) and then performing cyclic or linear-sweep voltammetry to measure the catalytic current as a function of potential in solutions containing relatively high concentrations (*i.e.*, 0.1 to 1 M) of acid.^13–18^ By focusing on the foot of the voltammetric wave, where ohmic drop and mass-transport limitations are minimal, the Tafel slope and exchange current density (*J*_0_) can be readily estimated (for a well-characterized electrode), which are two important metrics for evaluating catalytic performance.^1^ As highlighted above in the low [H^+^] study, the morphology of the meniscus cell is unaffected by the presence of surface defects on the tens of nm scale, evidenced from *I*_lim_ (see Fig. 2b), *I*_dc_ (see Fig. S7†) and SEM observation (*e.g.*, see Fig. 2c and S8†). With this in mind, voltammetric SECCM scanning experiments (see Fig. 1a) were carried out at a pH of 1 (*i.e.*, [H^+^] = 100 mM), as is shown in the ESI, Movie S2.†

**Fig. 3:**
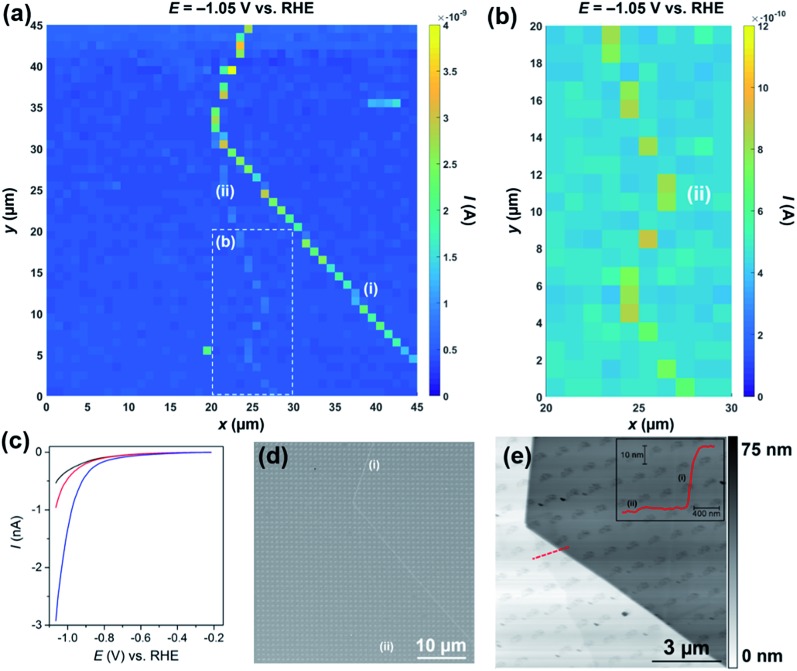
(a) 45 × 45 μm and (b) 10 × 20 μm [area indicated by the dashed white box in (a)] spatially resolved current maps (equipotential images) obtained at –1.05 V *vs.* RHE (see Movies S2 and S3† for full potential range). (c) Representative LSVs obtained from areas containing only basal plane (black trace, average of 1500 measurements), basal plane plus defect (i) (blue trace, average of 33 measurements) and basal plus defect (ii) (red trace, average of 14 measurements) of bulk MoS_2_. The following parameters were used in (a–c): [HClO_4_] = 100 mM, *ν* = 0.25 V s^–1^, *E*_b_ = +0.05 V, *r*_a_ = 220 nm and *r*_b_ = 110 nm. (d) SEM and (e) AFM topographical images of the scan area. Inset in (e) is an AFM line profile of the area indicated by the dashed red line. Major and minor surface defects are labelled as (i) and (ii), respectively.

The movie consists of 341 images (*i.e.*, 1 image every 2.5 mV), and each pixel (2025 in total) represents an individual LSV in the 45 × 45 μm scan area. A spatially resolved equipotential image (*i.e.*, current “map”), taken at –1.05 V *vs.* RHE is shown in Fig. 3a. Again, it is clear that the activity of the basal plane surface is relatively uniform (*i.e.*, the dark blue areas in Fig. 3a), and that the two surface defects [labelled (i) and (ii) in Fig. 3a] evident in the current map, give rise to enhanced catalytic currents in the applied potential range (see Movie S2†). Defect (i) produces a larger catalytic current than (ii), which is due to the former being much larger than the latter, as investigated in detail below. The enhanced activity of defect (ii) relative to the basal plane is more obvious in Fig. 3b and Movie S3,† which focuses on a 10 × 20 μm area of the surface, starting from *x* = 20 μm, *y* = 1 μm. Average LSVs extracted from pixels located on the basal plane, defect (i) and defect (ii) are shown in Fig. 2c; clearly, the HER catalytic activity increases in the order basal plane < (ii) ≪ (i), as highlighted above. Again, it should be emphasized that the red and blue *I*–*E* traces in Fig. 3b arise from predominantly basal plane plus a small area (estimated to be <10% of the total meniscus cell area, explored below) of edge plane.

The nature of the surface defects described above [(i) and (ii) in Fig. 3a and b] were investigated with a combination of SEM (see Fig. 3d) and AFM (see Fig. 3e). Both defects (i) and (ii) can be clearly seen with SEM (see Fig. 3d), particularly at higher magnifications, as shown in Fig. S12.† Both defects are also evident in the AFM topographical scan, as shown in Fig. 3e, and, as alluded to above, defect (i) is much larger than (ii), confirmed by AFM line profile scanning (see inset). Defect (i) is a large step (approx. 40 nm in height), likely made up of tens of MoS_2_ layers (theoretical thickness of monolayer MoS_2_ = 0.68 nm).^53^ Defect (ii) on the other hand is much smaller (approx. 2 nm in height), likely made up of a few (2 or 3) MoS_2_ layers. It is also worth noting that, as mentioned above, the individual droplet footprints are evident in the AFM topographical image, again implying that the voltammetric protocol employed during the electrochemical scanning (*i.e.*, cathodic sweeping) causes a physical (structural) change to the MoS_2_ surface. This exemplifies a key advantage of SECCM compared to other electrochemical imaging techniques for this type of application. For example, the study of this sample in this detail by scanning electrochemical microscopy (SECM) would be close to impossible: the sample would have to be fully immersed in the electrolyte solution, would be changing during the scanning protocol (voltammetric scan and physical motion of the tip) and, further, a small enough area of the surface would need to be electrically connected and masked off to avoid the significant sample resistive effects that were highlighted above. In contrast, SECCM investigates a series of very small and fresh surface regions.

**Fig. 4:**
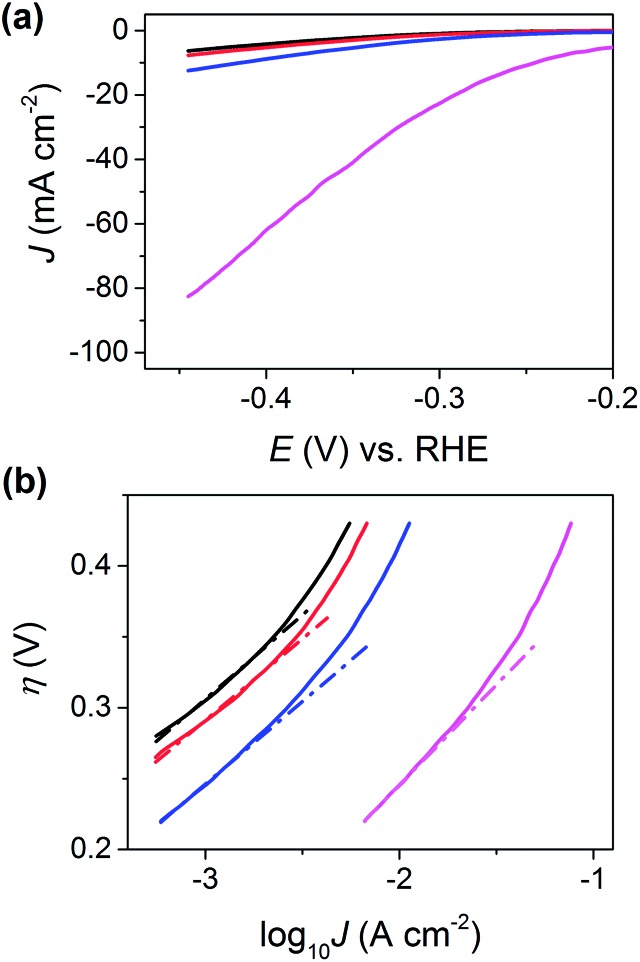
(a) LSVs (area normalized) and (b) Tafel plots obtained from MoS_2_ basal plane (black trace), MoS_2_ basal plane plus defect (i) (blue trace), MoS_2_ basal plus defect (ii) (red trace) and MoS_2_ edge plane (pink trace, estimated as described in the main text). The slopes and intercepts of the dashed lines shown in (b) were used to estimate the Tafel slope and *J*_0_, respectively. The following parameters were used to collect these data: [HClO_4_] = 100 mM, *ν* = 0.25 V s^–1^, *E*_b_ = +0.05 V, *r*_a_ = 220 nm and *r*_b_ = 110 nm.

The area of the surface probed by the meniscus cell (*i.e.*, the active electrode area) was estimated to be just 1.6 × 10^–9^ cm^2^ from the droplet footprints characterized by SEM (see Fig. S12†) and AFM (see Fig. 3e). Furthermore, by assuming that the major step [defect (i) in Fig. 3] transverses the minor axis of the elliptical droplet cell (*i.e.*, the length of the step is equal to *r*_b_) and has a height of 40 nm (derived from AFM, see Fig. 3e), its area was estimated to be 1.4 × 10^–10^ cm^2^. For clarity, the relative areas of the basal and edge planes are shown schematically in the ESI, Fig. S13.† The *I*–*E* response of the edge plane was estimated by subtracting the basal plane response (*i.e.*, the black trace in Fig. 3c) from that of defect (i) (*i.e.*, the blue trace in Fig. 3c). With these data, the LSVs were replotted in terms of current density (*J*), focusing on the potential range spanning from –0.20 to –0.48 V *vs.* RHE, as shown in Fig. 4a. Evidently, the estimated current density achieved at the edge plane (*i.e.*, pink trace in Fig. 4a) is more than an order of magnitude higher than that achieved at the basal plane (*i.e.*, black trace in Fig. 4a), in the investigated potential range.

In order to estimate Tafel slopes and *J*_0_, the data in Fig. 4a were replotted as *η*(*E* – *E*_RHE_)*vs.* log_10_ *J*, as shown in Fig. 4b. The Tafel slope associated with the basal plane, and basal plane plus defects (*i.e.*, black, red and blue traces in Fig. 4b) is approximately 120 mV per decade, which is consistent with the Volmer reaction (eqn (1)) being the rate determining step, although this conclusion should be treated with caution, as the Tafel slope alone is insufficient to determine the specific mechanism of the HER.^1,29,54^ The reported Tafel slope associated with MoS_2_ catalysts varies depending on the nature of the material (*i.e.*, synthesis conditions and support),^1,27^ although 120 mV per decade is consistent with a number of studies.^21,25,47^ Through extrapolation of the linear Tafel region, *J*_0_ was estimated to be 2.5 × 10^–6^ A cm^–2^, for the basal plane alone, which is comparable to the value reported by Yu and co-workers,^47^ who calculated a *J*_0_ of 1.1 × 10^–6^ A cm^–2^ for a (bottom-contacted) monolayer MoS_2_ film on a GC support. This clearly demonstrates that the basal plane of bulk MoS_2_ is just as active (if not slightly more active) than monolayer (basal plane) MoS_2_ if the electrical connection is made through top-contact. It is worth noting, that while the *J*_0_ of the basal plane of MoS_2_ is orders-of-magnitude lower than that of polycrystalline Pt (∼3 × 10^–3^ A cm^–2^), it is comparable to other polycrystalline transition metals, such as Co (∼5 × 10^–6^ A cm^–2^), Ni (∼6 × 10^–6^ A cm^–2^), Cu (∼4 × 10^–6^ A cm^–2^) and Au (∼4 × 10^–6^ A cm^–2^).^55^

Due to the requirement to minimize the overall time required to complete the voltammetric SECCM protocol (*i.e.*, high scan rate over minimal potential range), and measure currents over a large dynamic range (sub pA to nA, see Fig. 3c), the range of *J* over which the Tafel slope can be estimated is rather narrow in Fig. 4b. Thus, to confirm that the Tafel slope and *J*_0_ values are accurate, an additional experiment was carried out on a fresh sample of MoS_2_ (basal plane) at a much slower voltammetric scan rate (7.5 mV s^–1^), with much higher current sensitivity and potential resolution (0.3 mV per point), as shown in Fig. S14.† The Tafel slope and *J*_0_ were confirmed to be ∼120 mV per decade and ∼2.5 × 10^–6^ A cm^–2^, estimated from *J* data spanning 4 orders of magnitude.

It is worth noting that while SECCM has allowed the unambiguous determination of the *J*_0_ of the basal plane of bulk MoS_2_, it does not indicate on the nature of the active catalytic site. As alluded to in the introduction, and elucidated in a number of recent studies,^18,22–24^ point defects such as sulfur vacancies are likely responsible for the HER catalytic activity of the 2H basal plane. So while the basal plane voltammetric response (*e.g.*, see Fig. 3 and 4) has certainly been isolated from regions of the surface free of edge plane (*vide supra*), the probed areas undoubtedly contain point defects (*e.g.*, sulfur vacancies) which could contribute significantly to the observed catalytic activity.

The Tafel slope associated with the edge plane (*i.e.*, pink trace in Fig. 4b) is slightly higher than the basal plane at ∼130 mV per decade and *J*_0_ was estimated to be ∼1 × 10^–4^ A cm^–2^. Although this value should be taken *cum grano salis* due to the number of assumptions required to estimate the “area” of the step, it is clear that the edge plane of MoS_2_ (2H phase) is a much more active catalyst for the HER than the basal plane.^13–20^ It is also worth noting that edge plane MoS_2_ (2H phase) is still significantly less active than polycrystalline Pt (*J*_0_ ≈ 3 × 10^–3^ A cm^–2^)^55^ or nanostructured Pt-catalysts, which typically have reported *J*_0_ values in the range of 4 × 10^–4^ to 0.4 A cm^–2^.^1^

**Fig. 5:**
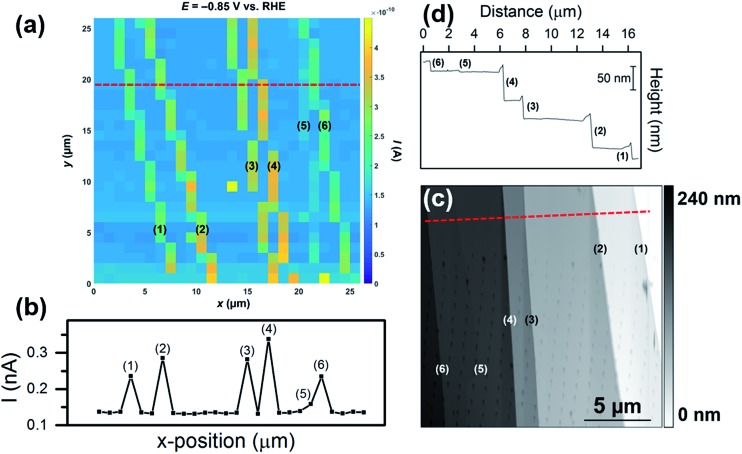
(a) 26 × 26 μm spatially resolved current map (equipotential image) obtained at –0.85 V *vs.* RHE (see Movie S4† for full potential range). Surface current line profile (surface current at –0.85 V *vs.* RHE *versus x*-position) taken from the dashed red line indicated in (a). The following parameters were used in (a) and (b): [HClO_4_] = 100 mM, *ν* = 0.25 V s^–1^, *E*_b_ = +0.05 V, *r*_a_ = 220 nm and *r*_b_ = 110 nm. (c) 18 × 18 μm AFM topographical scan taken of the scan area in (a). (d) AFM line profile of the area indicated by the dashed red line in (c). Each of the surface defects (steps) are labelled (1) to (6).

In the final set of experiments, the influence of the morphology of surface defects (*i.e.*, step size/area) on the enhancement in catalytic current was further investigated. A voltammetric SECCM movie, obtained in an area of MoS_2_ with multiple surface defects of differing sizes is shown in the ESI, Movie S4.† The movie consists of 301 images (*i.e.*, 1 image every 2.5 mV), and each pixel (676 in total) represents an individual LSV in the 26 × 26 μm scan area. A spatially resolved equipotential image (*i.e.*, current “map”), taken at –0.85 V *vs.* RHE is shown in Fig. 5a. Evidently, while the activity of the basal plane surface is relatively uniform (*i.e.*, the light blue areas in Fig. 5a), there are 6 surface defects [labelled (1) to (6), SEM image of the scan area is shown in Fig. S15a†] which show elevated currents, indicating enhanced HER catalytic activity. The magnitude of the catalytic current enhancement (and hence, the relative increase in activity compared to the basal plane) varies from defect-to-defect, which is obvious from a line-scan of substrate current, as shown in Fig. 5b (average LSVs extracted from each area are also shown in Fig. S15b†). The relative increase in activity (over the basal plane) is proportional to the height of the steps, confirmed by AFM topographical imaging (see Fig. 5c) and AFM line profile scanning (see Fig. 5d). By comparing Fig. 5b and d, it is clear that there is a strong correlation between the apparent activity and active edge plane area, with the measured catalytic current increasing in the order (5) < (6) ≈ (1) < (3) ≈ (2) < (4), while the step height increases in the order (5) < (6) ≈ (1) < (3) < (2) < (4). This again confirms that the measured catalytic activity increases with an increase in the proportion of edge plane to basal plane in the area probed by the SECCM droplet (meniscus) cell.

## 4. Conclusions

The electrocatalytic activity of bulk MoS_2_ for the HER was investigated at the nanoscale using voltammetric SECCM in combination with SEM and AFM in a *correlative multi-microscopy approach*. The basal plane of bulk MoS_2_ (from natural molybdenite) was found to support the HER, with catalytic activity significantly better than GC, contrary to macroscopic electrochemical measurements made with this material. Spatially-resolved LSV measurements were performed to construct electrochemical flux movies of equipotential images (current maps) across a wide potential range, and by correlating the electrochemical activity maps with complementary (structural) information from SEM and AFM, it has been unequivocally shown that the presence of surface disorder (*i.e.*, defects, steps or crevices) enhances the kinetics of the HER on bulk MoS_2_. Semi-quantitative treatment of the voltammetric data revealed that the basal plane of bulk MoS_2_ has a Tafel slope and *J*_0_ of ∼120 mV per decade and 2.5 × 10^–6^ A cm^–2^ (previously unreported, comparable to polycrystalline Co, Ni, Cu and Au), respectively, while the edge plane possesses a similar Tafel slope (∼130 mV per decade) and an estimated *J*_0_ of ∼1 × 10^–4^ A cm^–2^. Finally, cathodic polarization of MoS_2_ in acidic media was found to clean (revealed by time-dependent WCA measurements) and physically alter the structure of the surface (revealed by SEM and AFM). Overall, this study, which would be close to impossible with other scanning probe techniques such as SECM (due to sample aging upon immersion and ohmic drop), further demonstrates the great versatility of SECCM for the nanoscale imaging (and structure–function correlation) of nanostructures with both conventional (*e.g.*, particles on conductive supports) and unconventional (*e.g.*, resistive materials such as bulk MoS_2_) substrates.
