# Supplementary material for: Electrochemical maps and movies of the hydrogen evolution reaction on natural crystals of molybdenite (MoS2): basal vs. edge plane activity
Source: Chem Sci. 2017 Jul 26;8(9):6583–93. doi: 10.1039/c7sc02545a (PMC5627349; doi:10.1039/c7sc02545a)
Supplement: Supplementary file 1 [file SC-008-C7SC02545A-s001.pdf]

Electronic Supplementary Material (ESI) for Chemical Science.
This journal is © The Royal Society of Chemistry 2017

Supporting Information (SI) for:

Electrochemical Maps and Movies of the Hydrogen Evolution Reaction on Natural Crystals of Molybdenite (MoS_2): Basal vs. Edge Plane Activity

Cameron L. Bentley[a,*], *Minkyung Kang*[a], *Faduma M. Maddar*[a], *Fengwang Li*[b], *Marc Walker*[c], *Jie Zhang*[b] *and Patrick R. Unwin*[a,*]

[a]Department of Chemistry, University of Warwick, Coventry CV4 7AL, U.K.

[b]School of Chemistry and Australian Research Council Centre of Excellence for Electromaterials Science, Monash University, Clayton, Vic 3800, Australia

[c]Department of Physics, University of Warwick, Coventry CV4 7AL, U.K.

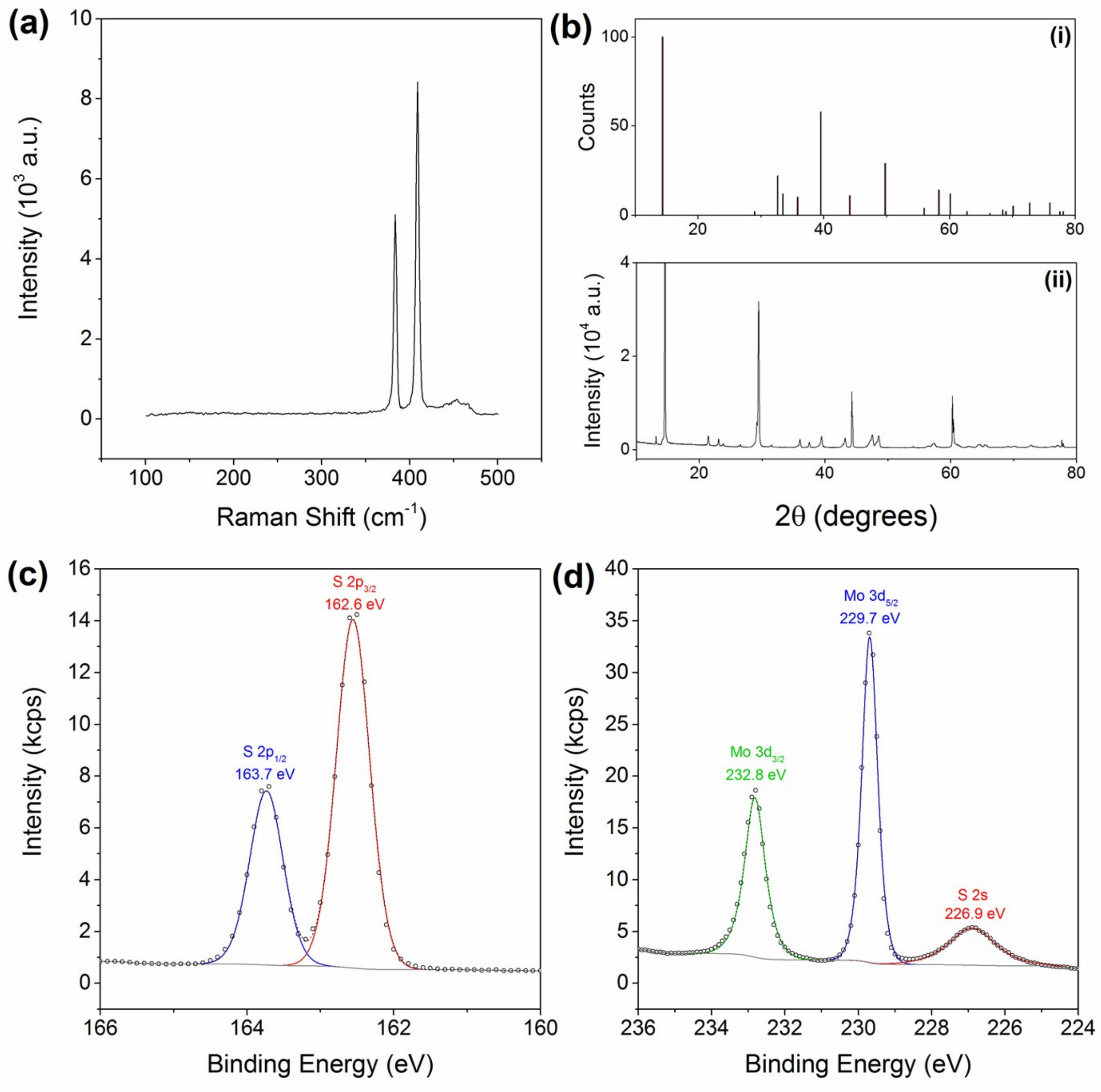

Figure S1. (a) Raman, (b) XRD [(i) 2H MoS2 PDF#37-1492 and (ii) experimental] and XPS [(c) S 2p and (d) Mo 3d] spectra obtained and XPS spectra obtained from a freshly exfoliated sample of bulk MoS_2. Experimental (○) and fitted (---) data are shown in (c) and (d).

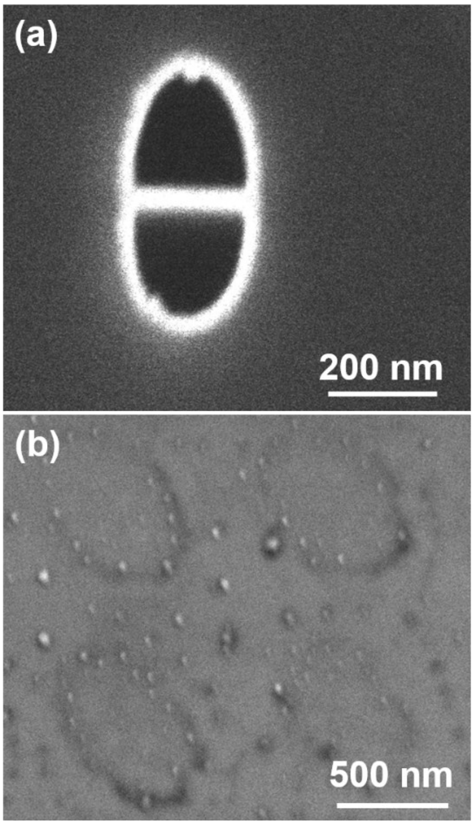

Figure S2. Scanning electron micrographs showing (a) the pulled end of a nanopipet probe ($r_a \approx 250$ nm and $r_b \approx 130$ nm) used during SECCM and (b) an example of droplet footprints left on the surface of a MoS_2 crystal after voltammetric SECCM scanning.

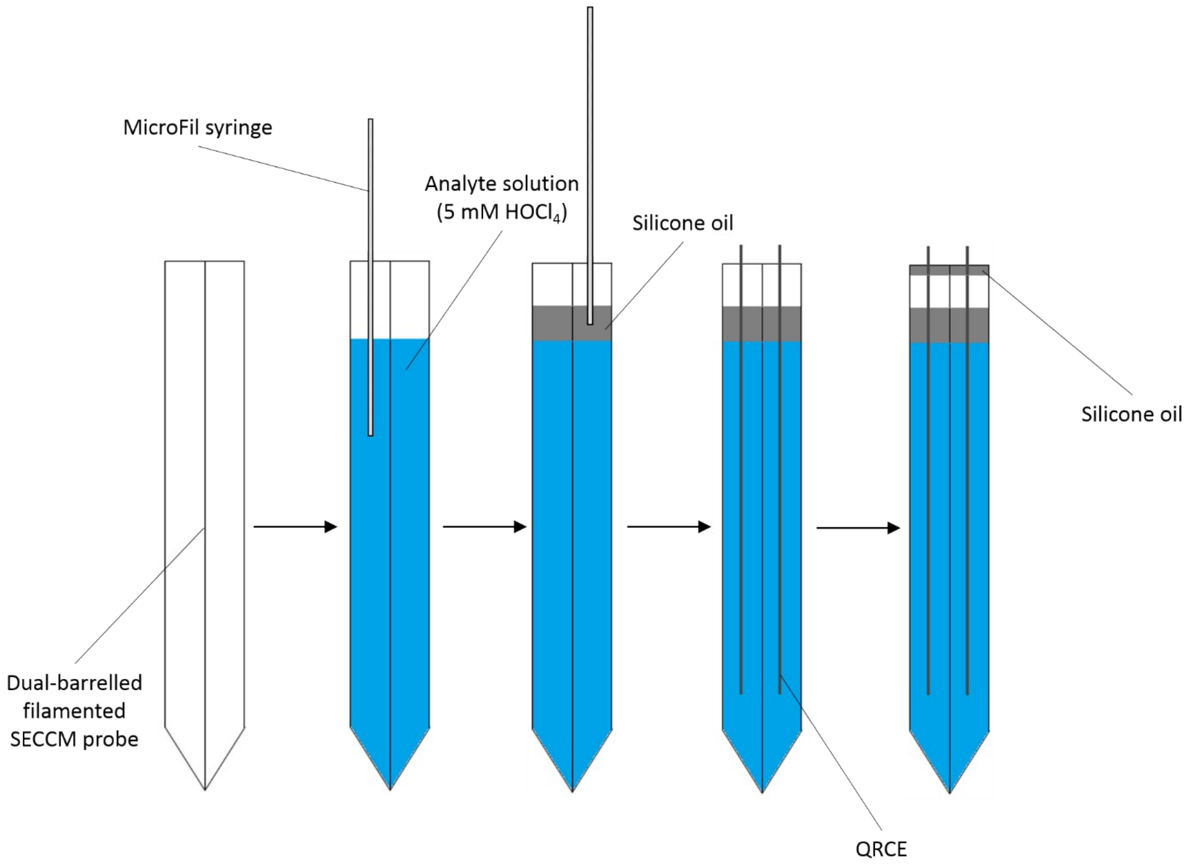

Figure S3. Schematic diagram showing the process used to fill the nanopipet probes employed during SECCM. A layer of silicone oil was added on top of the analyte solution to prevent the filamented probe from "drying out" during prolonged scanning.

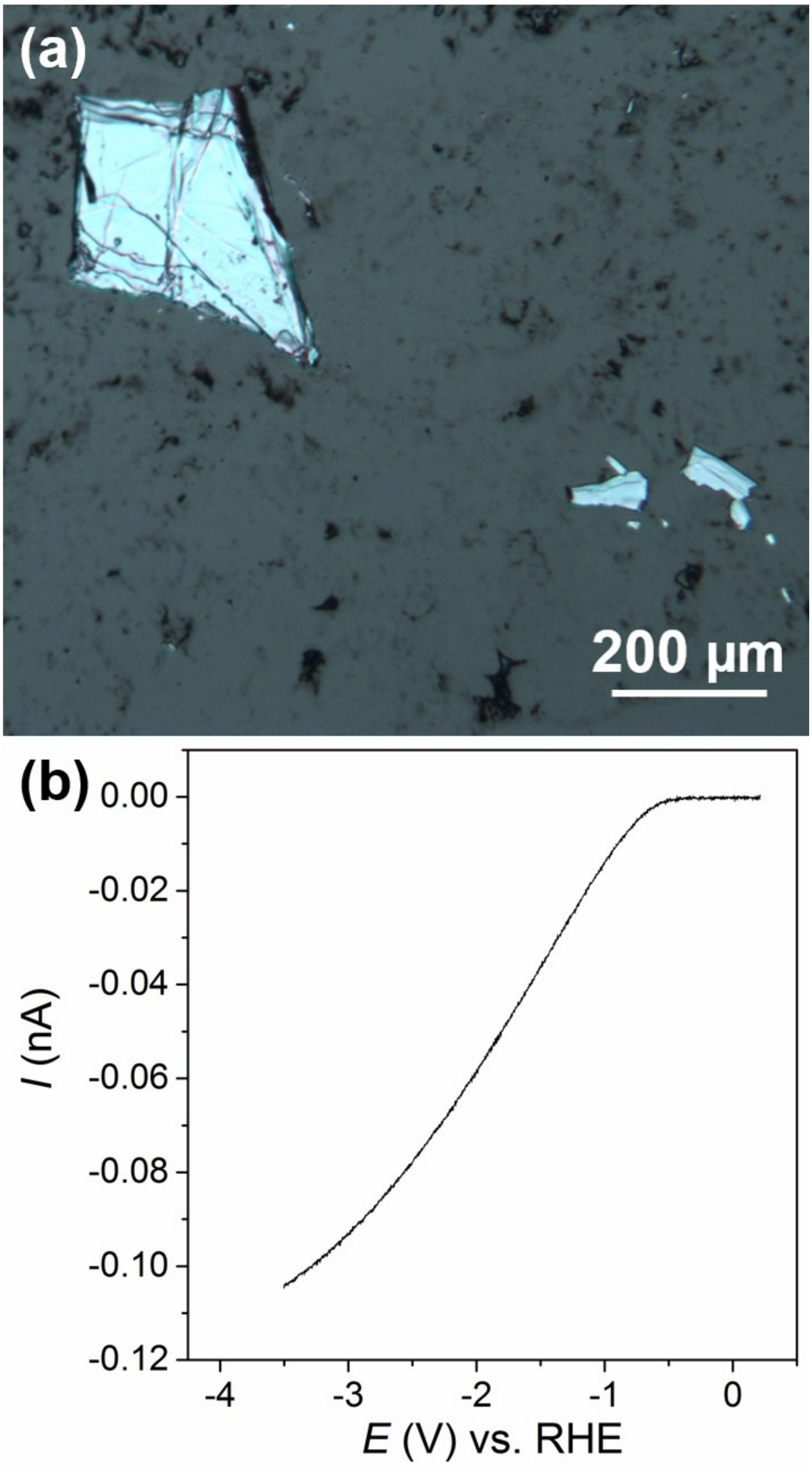

Figure S4. (a) Optical micrograph showing pieces of a MoS_2 crystal physisorbed on a GC substrate. (b) LSV (v = 1 V s^{-1}, E_{bias} = +0.1 V) obtained from 3 mM $HClO_4$ on the MoS_2/GC substrate shown in (a).

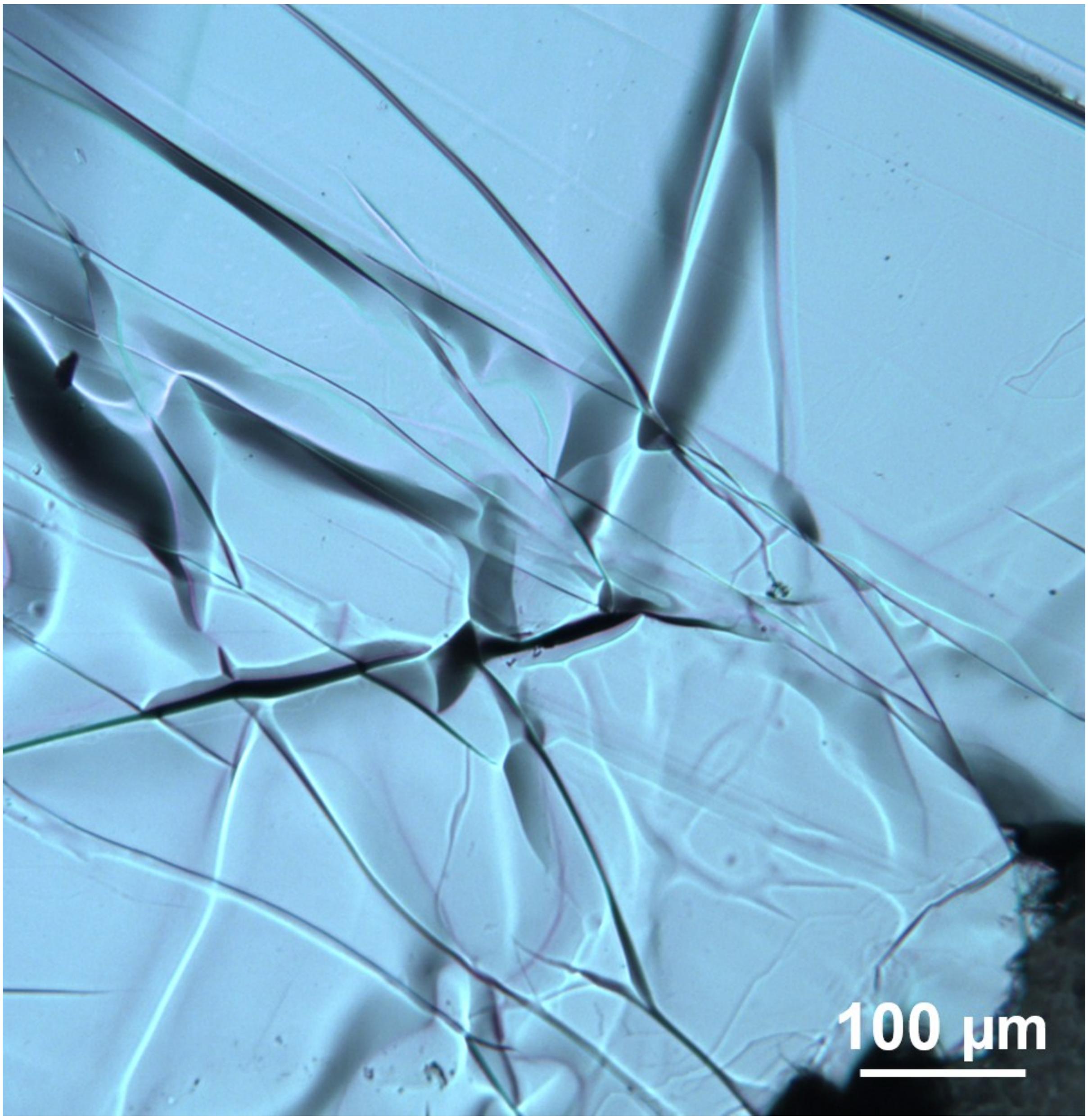

Figure S5. Optical micrograph of a bulk MoS_2 substrate after cleavage.

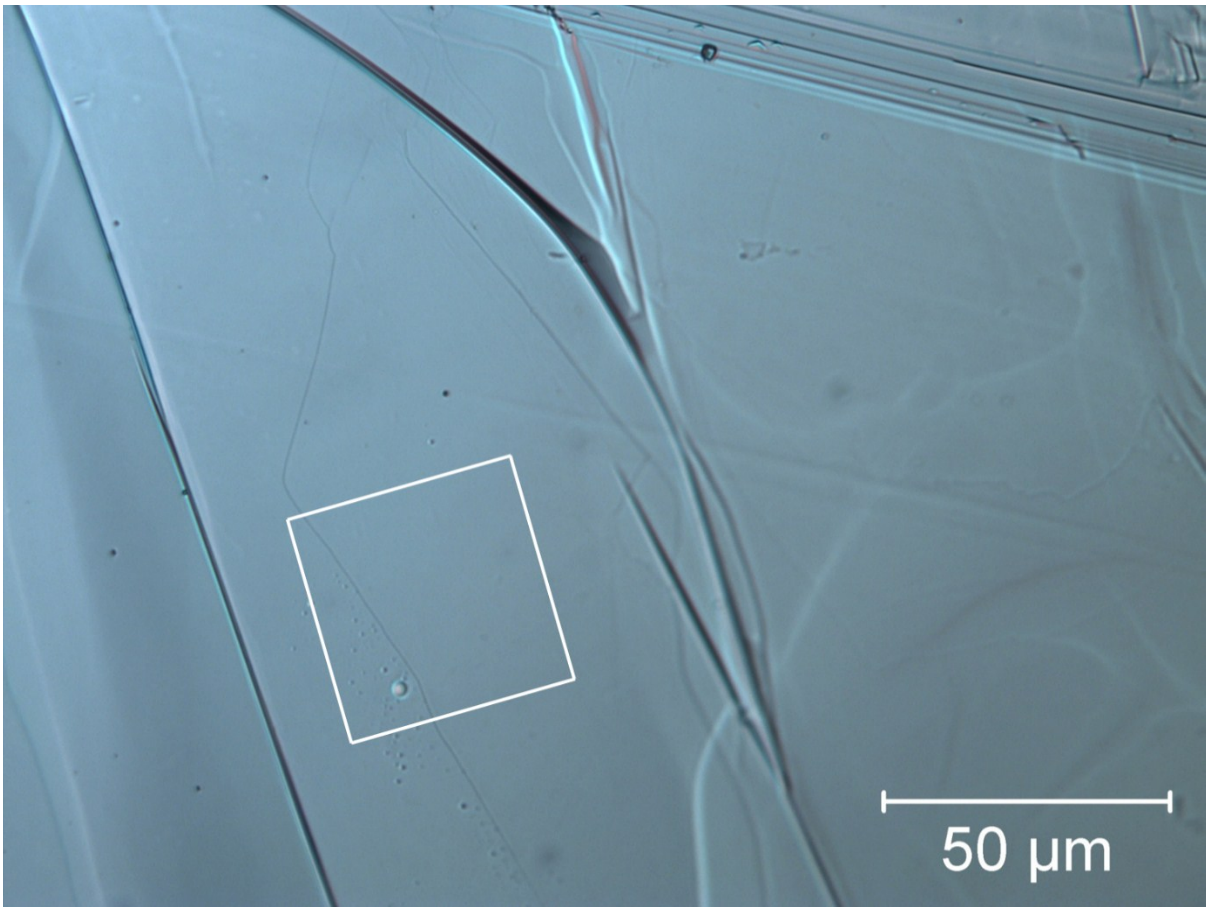

Figure S6. Optical micrograph showing the 40×40 µm area scanned by voltammetric SECCM, as shown in Figure 2a of the main text.

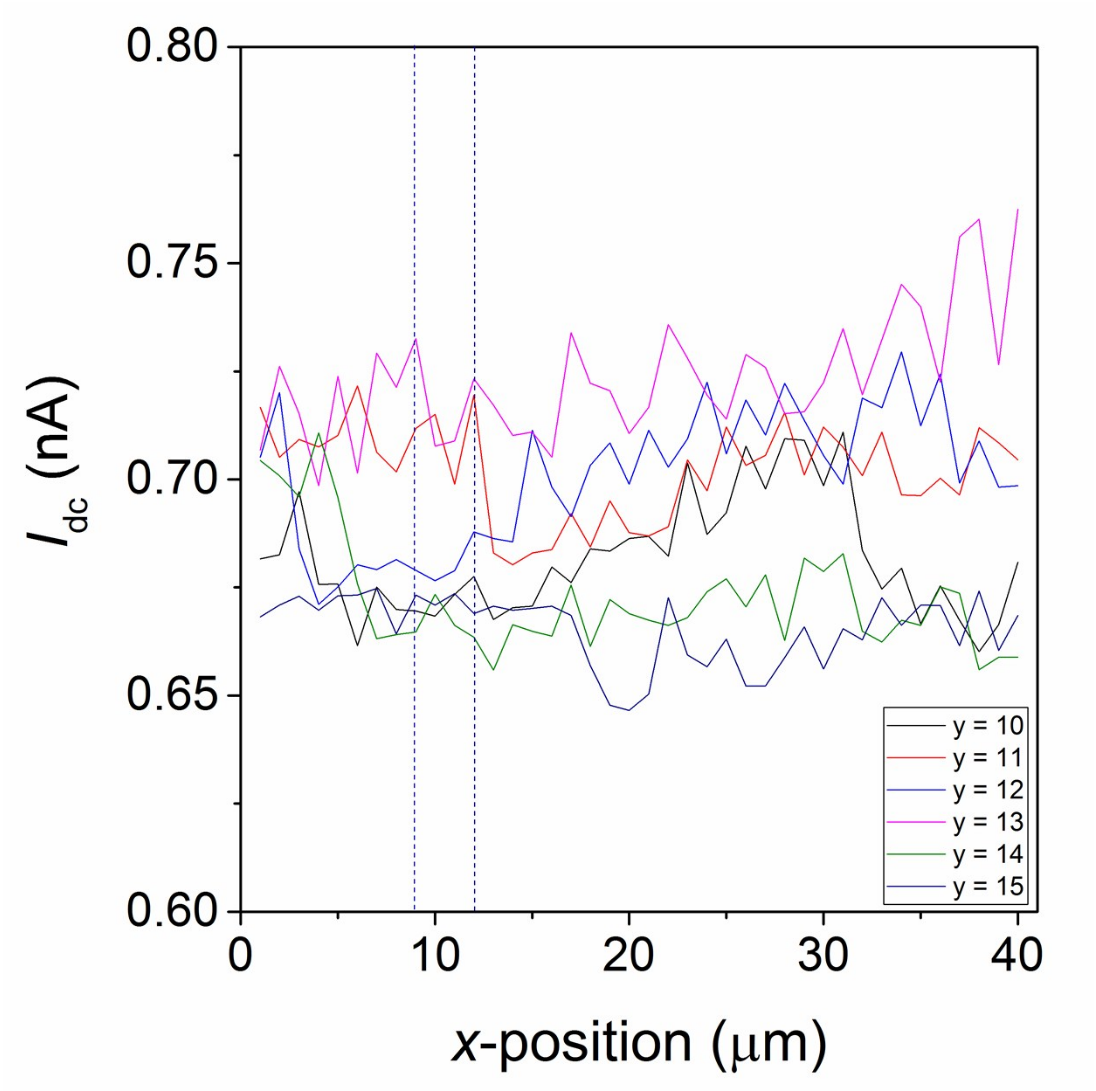

Figure S7. Line profiles (y = 10 to 15) of the *dc* ion conductance current (at –0.15 V vs. RHE) versus the x-position. The blue lines indicated on the plot delineate the area in which a major surface defect [defect **(i)**, see Figure 2 of the main text] is located.

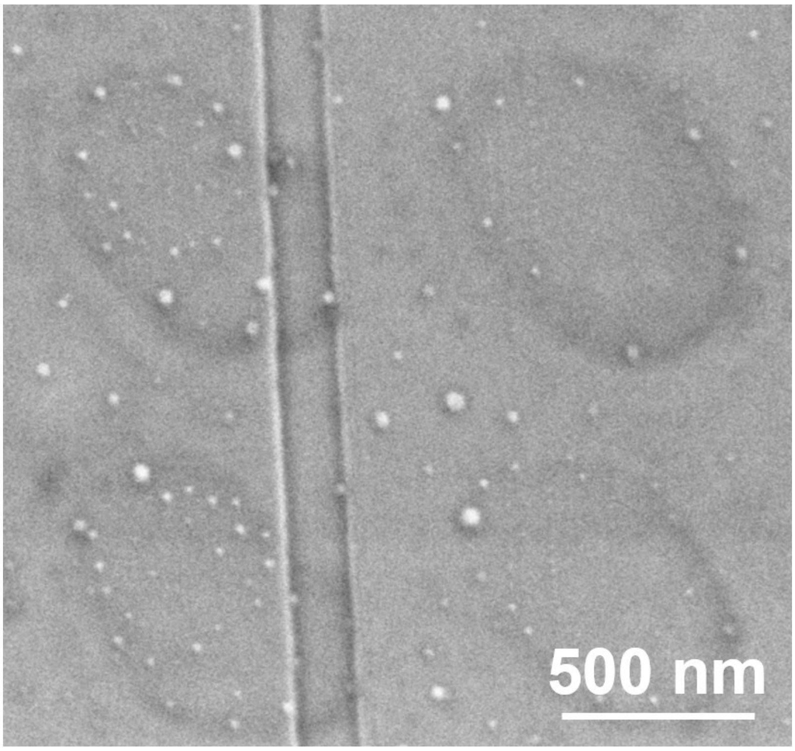

Figure S8. Scanning electron micrograph of droplet footprints left on the surface of bulk MoS_2 after voltammetric SECCM scanning, as shown in Figure 2a of the main text. Overlap of scanned areas with the major defect [defect (i) in Figure 2] is evident in this image.

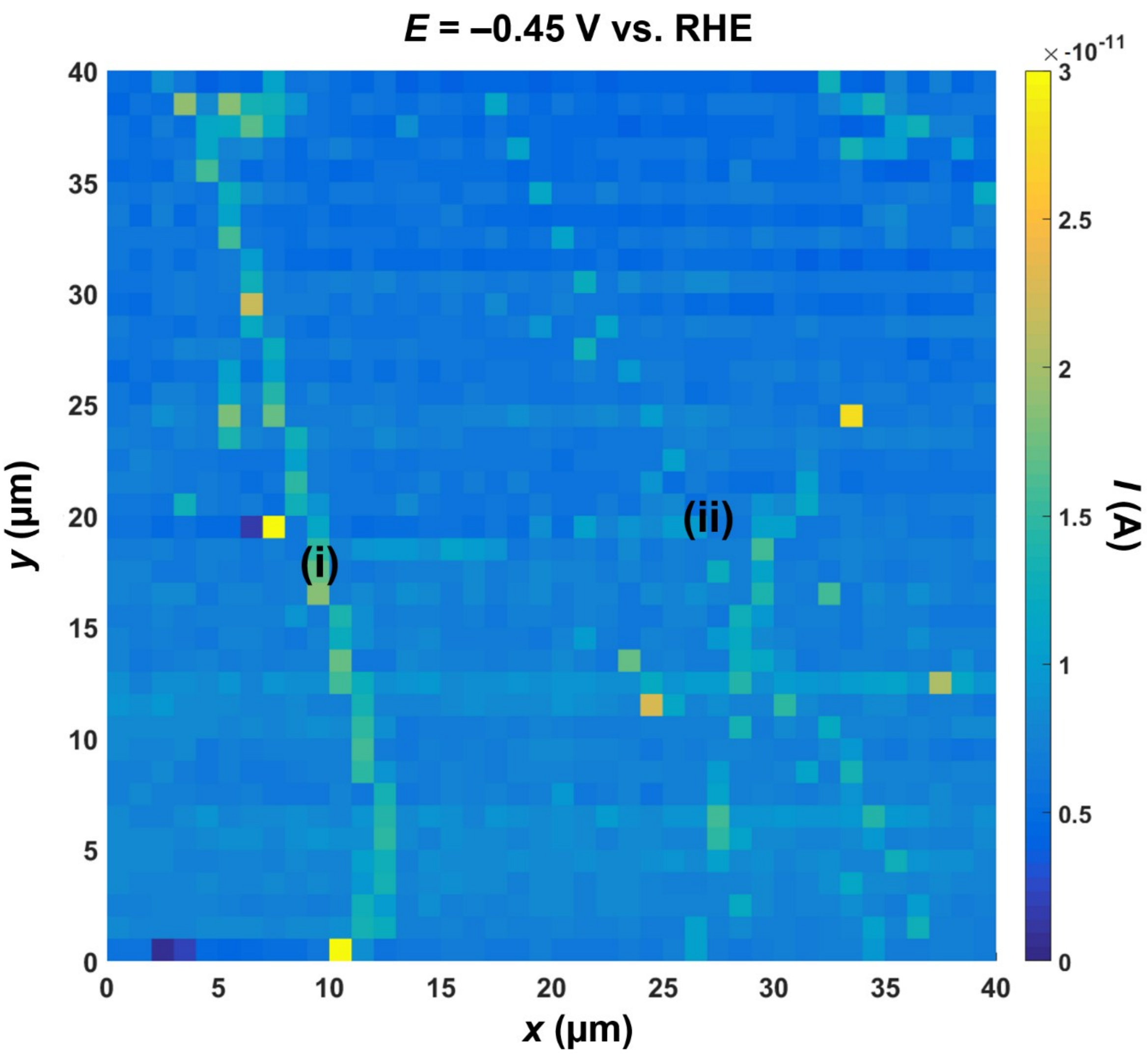

Figure S9. 40×40 µm spatially resolved current map (equipotential image) obtained at –0.45 V vs. RHE (see Movie S1 for full potential range). Major and minor surface defects are labelled as **(i)** and **(ii)**, respectively. The following conditions were used during the scan: $[HClO_4]$ = 5 mM, v = 0.5 V s^{-1}, E_b = +0.2 V, r_a = 275 nm and r_b = 125 nm.

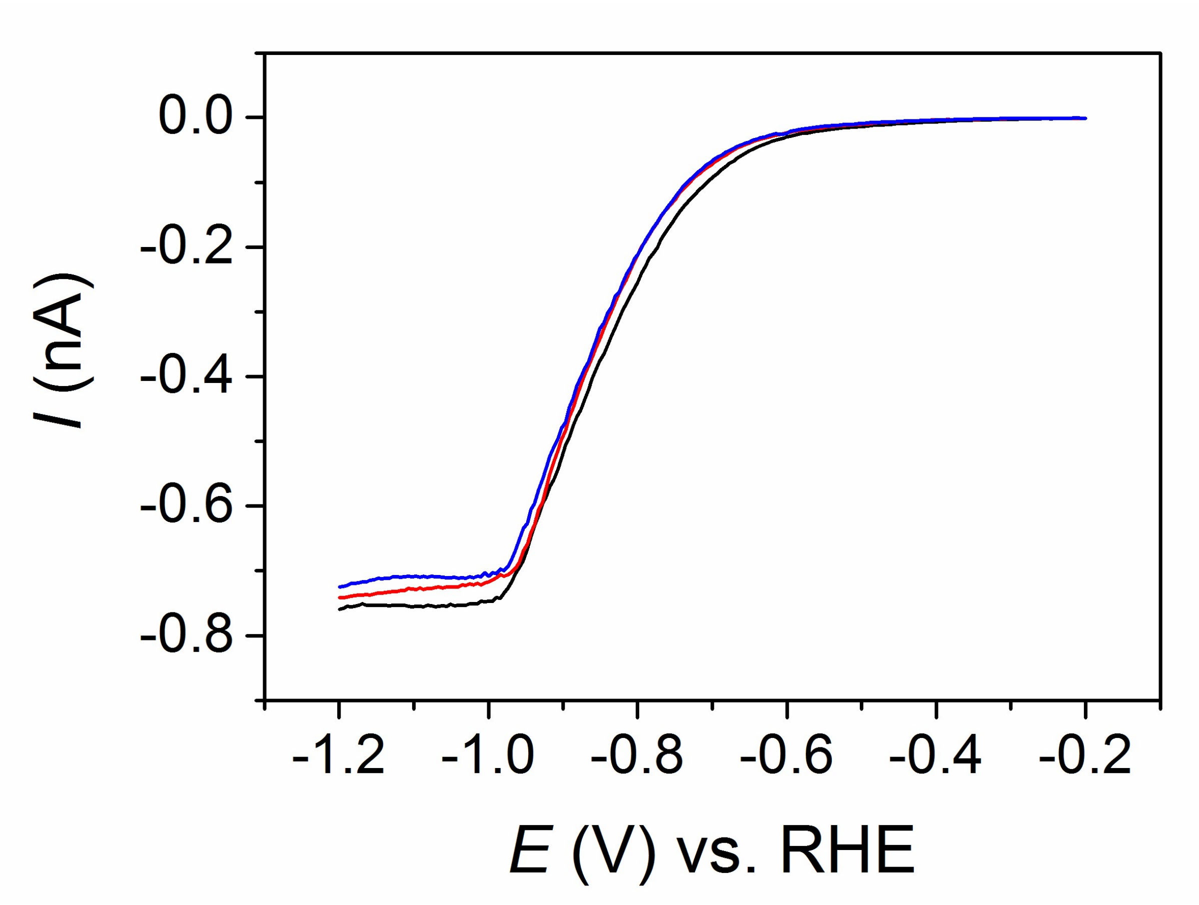

Figure S10. LSVs taken from the start (black trace, $y = 3$), middle (red trace, $y = 22$) and end (blue trace, $y = 39$) of the voltammetric SECCM scan shown in Figure 2 of the main text. Each LSV is the average of five measurements. The following conditions were used during the scan: [$HClO_4$] = 5 mM, $v = 0.5$ V s^{-1}, $E_b = +0.2$ V, $r_a = 275$ nm and $r_b = 125$ nm.

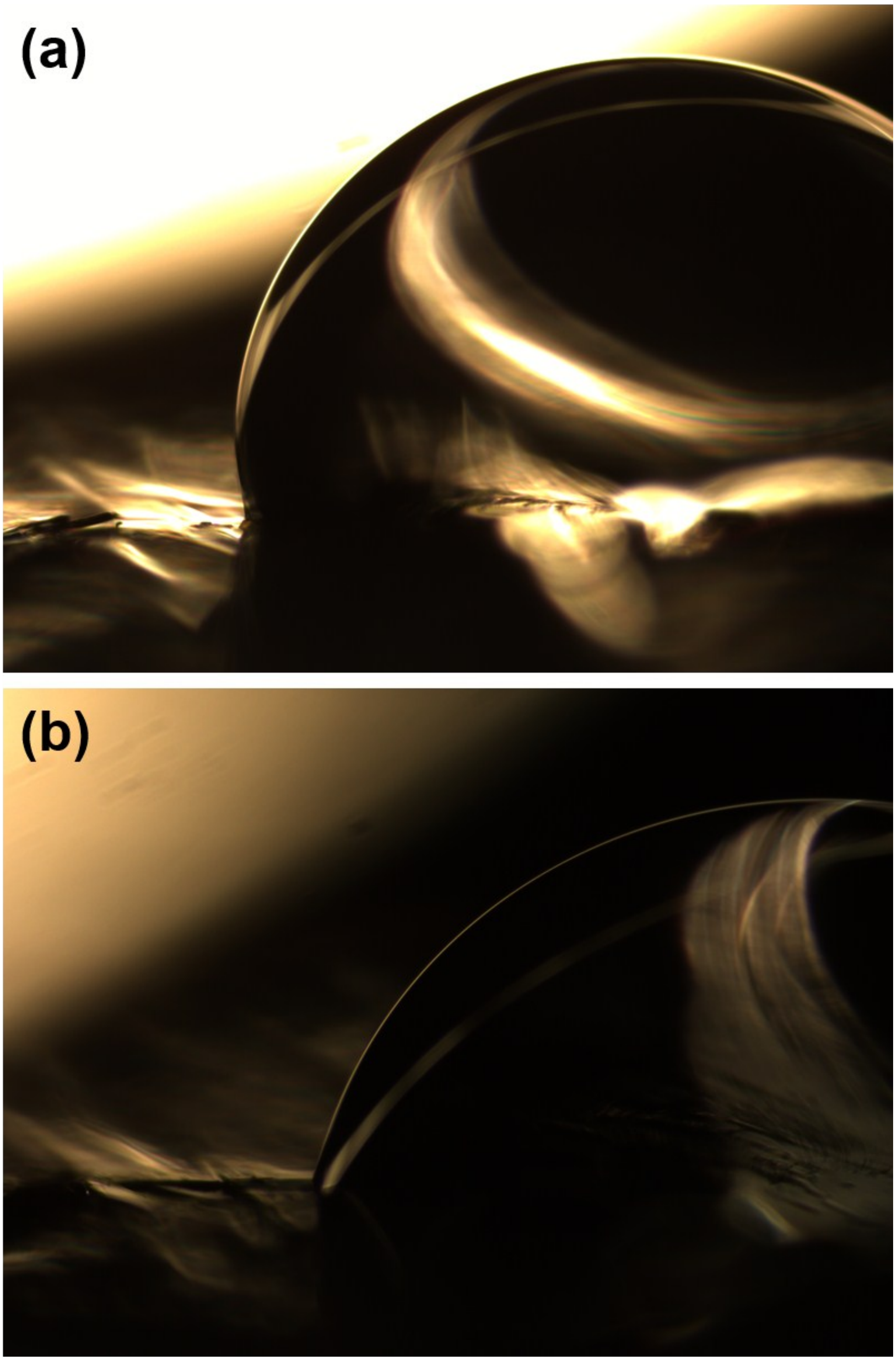

Figure S11. Images of a 10 μL water droplet atop an aged MoS_2 surface (a) before and (b) after voltammetric cycling between +0.6 and –1.9 V vs. RHE at a scan rate of 0.05 V s^{-1}. The WCA is 98º and 73º in (a) and (b), respectively.

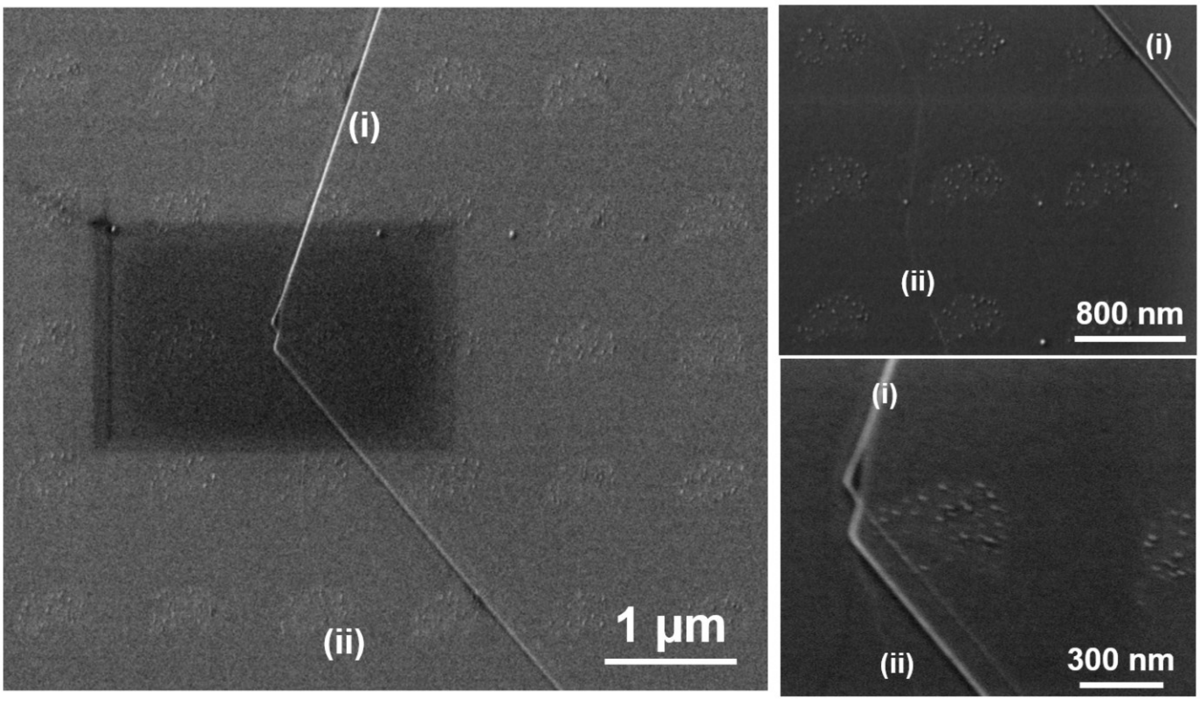

Figure S12. Scanning electron micrographs of the surface of bulk MoS_2 after voltammetric SECCM scanning, as shown in Figure 3a of the main text. Major and minor surface defects are labelled as (i) and (ii), respectively.

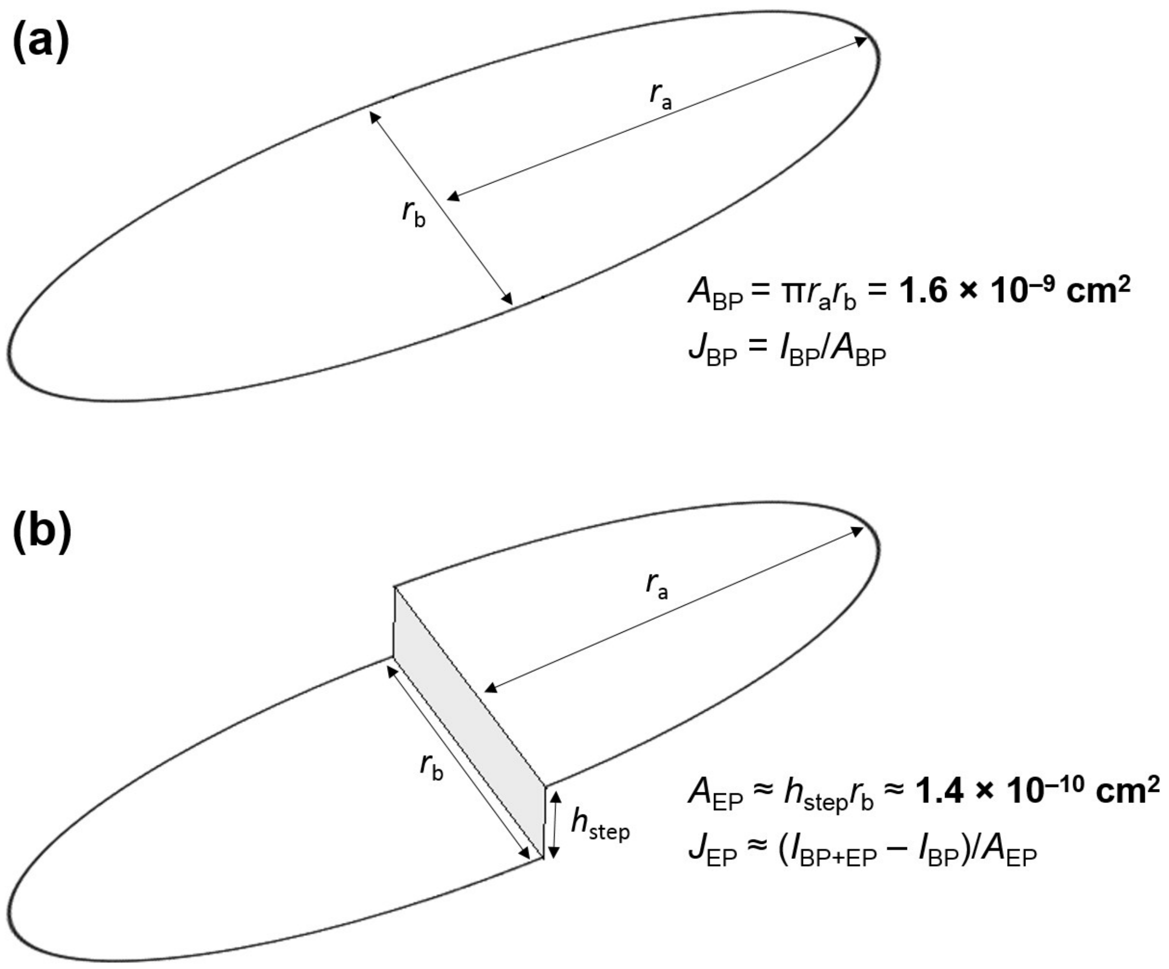

Figure S13. Schematic diagram showing the probed region of the SECCM droplet cell in an area containing (a) pure basal plane and (b) predominantly basal plane plus a surface defect. Also shown is the calculations for the active electrode area (*A*) and current density (*J*) associated with the basal plane (subscript BP) and edge plane (subscript EP). The area calculated in (b) corresponds to defect **(i)** in Figure 3 of the main text.

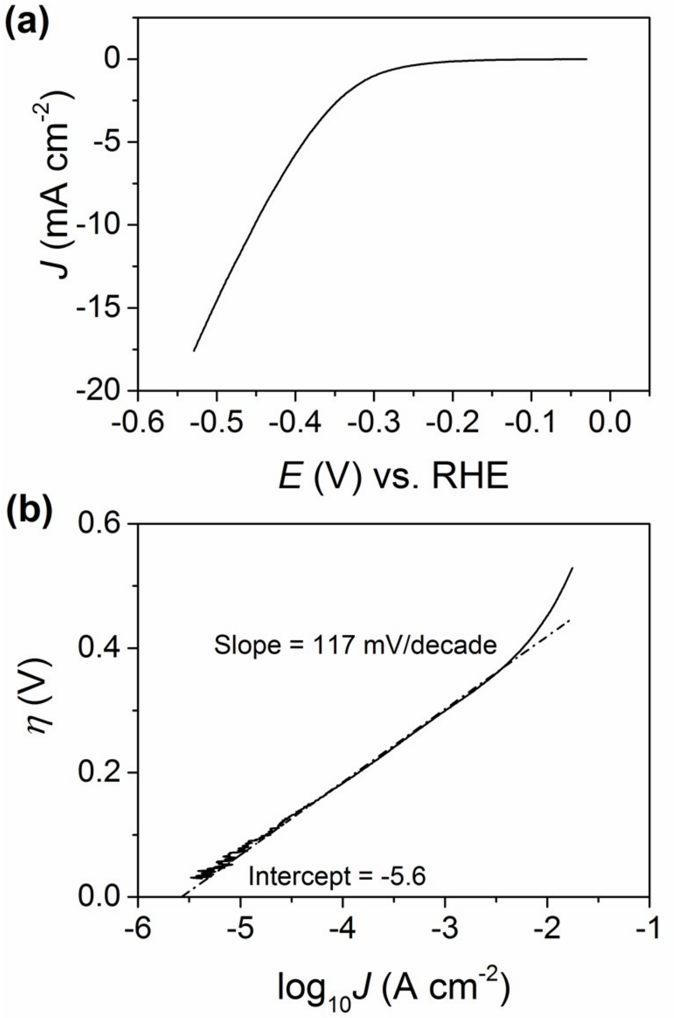

Figure S14. A (a) LSV (area-normalized) and (b) Tafel plot obtained from the HER on the MoS_2 basal plane (average of 222 measurements). The slope and intercept of the dashed line shown in (b) was used to estimate the Tafel slope and J_0, respectively (indicated on the plot). The following parameters were used to collect these data: $[HClO_4]$ = 100 mM, v = 7.5 mV s^{-1}, E_b = +0.05 V, r_a = 250 nm and r_b = 130 nm.

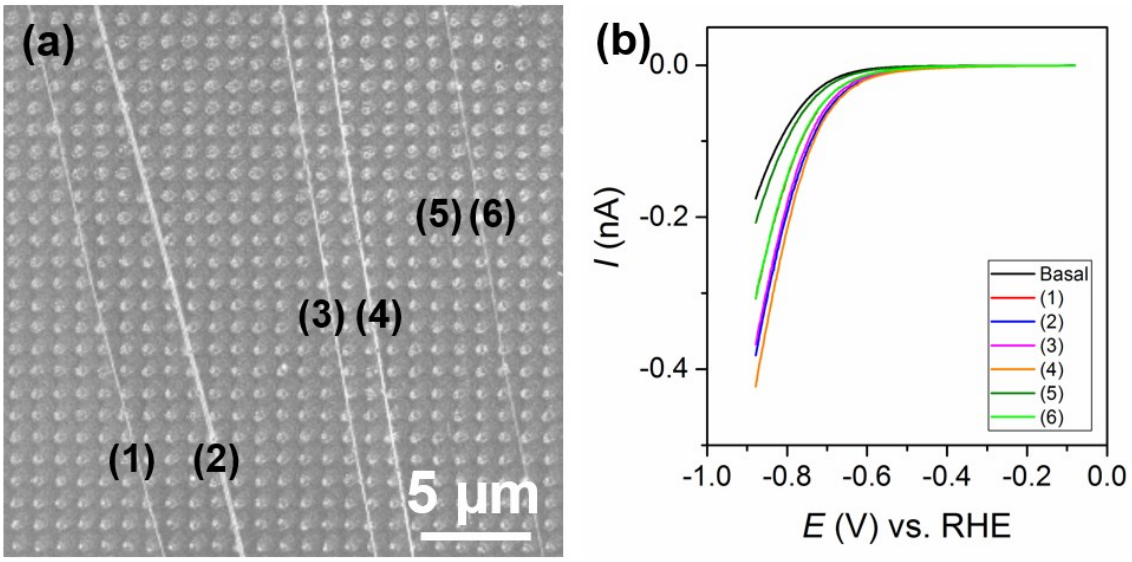

Figure S15. (a) A scanning electron micrograph of the surface of bulk MoS_2 after voltammetric SECCM scanning (26×26 μm area), as shown in Figure 5a of the main text. (b) Representative LSVs obtained from the basal plane and defects **(1)** to **(6)**, as indicated in (a). The following parameters were used in (b): [$HClO_4$] = 100 mM, v = 0.25 V s^{-1}, E_b = +0.05 V, r_a = 220 nm and r_b = 110 nm.
